# *Inocutis levis* Mycelial Extract WYS Exerts Anti-Proliferative and Pro-Apoptotic Effects on MCF-7 Cells via EGFR/MEK/ERK Modulation and G2/M Arrest

**DOI:** 10.3390/foods15183199

**Published:** 2026-09-10

**Authors:** Shaojun Tang, Yan Shu, Jun Zhang, Lianlian Yan, Huajun Zhu, Shenglian Wu, Chenxia Shao, Jieli Mo, Xuning Liu, Ning Xu, Jun Xu

**Affiliations:** 1 Hunan Institute of Microbiology, Changsha 410009, China; hnswsw_tsj@163.com (S.T.); hnswsw_sy@163.com (Y.S.); hnswsw_zj@163.com (J.Z.); hnswsw_yll@163.com (L.Y.); hnswsw_zhj@163.com (H.Z.); hnswsw_wsl@163.com (S.W.); hnswsw_scx@163.com (C.S.); hnswsw_mjl@163.com (J.M.); hnswsw_lxn@163.com (X.L.); 2 Yuelushan Laboratory, Changsha 410125, China; 3 Institute of Hunan Edible Fungi, Changsha 410006, China

**Keywords:** *Inocutis levis*, functional food, breast cancer, macrofungus, cell cycle arrest, mitochondrial apoptosis, EGFR/MEK/ERK pathway

## Abstract

*Inocutis levis* (*I. levis*) is a rare edible and medicinal macrofungus with a traditional history of consumption as a dietary supplement and functional food raw material. However, the material basis and molecular mechanisms underlying its anti-breast cancer activity remain unclear. The present work aimed to systematically investigate the anti-proliferative activity of WYS, an active mycelial fraction isolated from *I. levis*, against MCF-7 breast cancer cells and to explore its underlying mechanisms. Firstly, 152 metabolites were putatively annotated in the WYS fraction via ultra-performance liquid chromatography–quadrupole time-of-flight mass spectrometry (UPLC-Q-TOF-MS) combined with database matching. Through an integrated strategy combining network pharmacology and multi-algorithm machine learning screening, four core metabolites (dihydroartemisinin, cortisol, cinnamic acid, and guanosine) and five hub targets (CDK1, EGFR, TOP2A, FGF2, and KIT) were prioritized. Furthermore, 100 ns molecular dynamics simulations and molecular docking predicted favorable binding affinities between the core metabolites and their cognate target proteins and predicted that the anti-breast cancer effect of WYS may be associated with the regulation of cell cycle progression, mitochondrial apoptosis, and the MAPK signaling pathway. Subsequent in vitro functional assays validated that WYS inhibited the proliferation of MCF-7 cells in both dose- and time-dependent fashions, triggered G2/M phase arrest and intrinsic mitochondrial apoptosis, and inhibited the EGFR/MEK/ERK signaling axis, exhibiting limited cytotoxicity toward normal HUVEC cells. In vivo, WYS dose-dependently attenuated xenograft tumor growth, and its modulatory effects on cell cycle, apoptosis, and related signaling pathways were consistent with the in vitro observations. This study provides the first evidence that *I. levis* mycelial fraction WYS modulates EGFR/MEK/ERK-related signaling and induces G2/M cell cycle arrest, thereby exerting anti-proliferative and pro-apoptotic effects on MCF-7 breast cancer cells, providing a scientific basis for further investigation of *I. levis* as a functional food.

## 1. Introduction

Breast cancer is the most frequently diagnosed malignancy in women worldwide, featuring high molecular heterogeneity, strong invasiveness and metastatic potential, which pose major challenges to clinical management [1]. Conventional therapeutic modalities (surgery, radiotherapy, chemotherapy, and targeted therapy) deliver moderate clinical benefits but are commonly constrained by adverse events, acquired drug resistance, and subtype-specific limitations [2,3]. Accordingly, exploring efficacious, low-toxicity multi-target natural products has emerged as a critical direction in anti-tumor research.

Macrofungi represent a valuable resource pool of natural anti-tumor bioactive substances. They biosynthesize structurally diverse secondary metabolites including flavonoids, triterpenes, polyphenols, lactones, steroids and alkaloids [4,5], endowing them with prominent multi-target regulatory advantages and favorable biosafety [6,7,8]. Representative studies show that *Ganoderma lucidum* extract suppresses breast cancer cell proliferation and induces apoptosis by modulating the PI3K/AKT/mTOR pathway [9], and *Phellinus igniarius* extract exerts dual anti-tumor effects against colitis-associated colorectal cancer through direct cytotoxicity and macrophage functional reprogramming [10]. Nevertheless, these findings from other fungal species cannot be directly generalized to I. levis due to interspecific differences, and its biological activity requires independent experimental verification.

*Inocutis levis* (*I. levis*) is a rare edible and medicinal macrofungus with significant developmental potential, taxonomically classified under the phylum Basidiomycota, class Agaricomycetes, order Hymenochaetales, family Hymenochaetaceae, and genus *Inocutis*. Its fruiting body is annual, sessile, corky to fibrous, usually growing singly on living or fallen broad-leaved trees from late summer to autumn, causing white rot of wood [11,12]. Taxonomic studies indicate that this species was initially assigned to the genus *Inonotus* and regarded as an important group of broad-sense poplar *Inonotus vannni*. With advances in phylogenetic research, it has been assigned to the genus *Inocutis*. Currently, over 12 species of the genus *Inocutis* have been reported globally, including nine species distributed in China, and its taxonomic status has been widely recognized by the academic community [13]. Within the genus *Inocutis*, *Inocutis tamaricis* has been relatively well studied, with confirmed antioxidant and anti-tumor activities [14,15]. As a rare medicinal fungus of the genus *Inocutis*, *I. levis* has long been used as an adjuvant treatment for tumors and diabetes in southern China, and modern pharmacological studies have preliminarily confirmed its diverse biological activities [16,17]. However, global research on *I. levis* remains highly limited; no systematic small-molecule metabolomic profiling, in-depth anti-breast cancer efficacy evaluation, or mechanistic investigation has been reported, which severely hinders the scientific understanding and development of this rare edible-medicinal fungus.

Traditional pharmacological studies mostly focus on the single-component and single-target model, which is difficult to fully reveal the multi-component, multi-target and multi-pathway synergistic regulatory network of natural products. Network pharmacology integrates systems biology, bioinformatics and computational pharmacology, enabling efficient prediction of drug–disease–target interactions, and has been widely applied in the study of various tumors such as breast cancer and liver cancer [18,19]. In breast cancer research, this technology is extensively used for mechanism elucidation of natural products: for instance, studies have confirmed that honokiol exerts anti-triple-negative breast cancer effects by regulating targets such as HSP90AA1 and AKT1, as well as pathways including PI3K-Akt and MAPK [20]. Similarly, research on L-mimosine also demonstrates its advantages in exploring multi-target regulatory mechanisms [21].

At present, most existing studies on *I. levis* focus on polysaccharide components, while the small-molecule secondary metabolites of *I. levis* and their anti-tumor activity remain largely unexplored. Accordingly, this study intends to systematically elucidate the material basis and molecular mechanism of *I. levis* against breast cancer. Following metabolite profiling via UPLC-Q-TOF-MS, we screened core candidate metabolites and hub targets through network pharmacology and multi-algorithm machine learning, and predicted their binding stability by molecular docking and dynamics simulation. The anti-tumor efficacy and mechanisms were further validated by in vitro cellular assays and in vivo xenograft experiments. This study not only provides scientific support for the development of *I. levis* as a functional food ingredient and a dietary adjuvant intervention for breast cancer but also establishes a replicable research paradigm for exploring active components from edible and medicinal macrofungi.

## 2. Materials and Methods

### 2.1. Materials

*I. levis* was collected and identified by the Hunan Institute of Microbiology (NCBI accession number: KU058398.1) and preserved in the China General Microbiological Culture Collection Center (preservation number: CGMCC No. 21934), Beijing, China. Human breast cancer MCF-7 cells and human umbilical vein endothelial (HUVEC) cells were purchased from the China Center for Type Culture Collection (CCTCC), Wuhan, Hubei, China.

### 2.2. Key Instruments and Reagents

UPLC-Q-TOF-MS system: AB Sciex Exion-QTOF X500R, AB Sciex, Framingham, MA, USA. Flow cytometer: FACSCalibur, BD Biosciences, San Jose, CA, USA. Laser scanning confocal microscope: TCS SP8, Leica Microsystems, Wetzlar, Germany. RPMI-1640 medium: Gibco, Thermo Fisher Scientific, Waltham, MA, USA. Fetal bovine serum: Biological Industries, Kibbutz Beit Haemek, Israel. MTT reagent, Annexin V-FITC/PI apoptosis kit, PI cell cycle kit, JC-1 kit: Beyotime Biotechnology, Shanghai, China. All antibodies are from Cell Signaling Technology, Danvers, MA, USA.

### 2.3. Preparation of WYS

A 10 L liquid culture medium for *I. levis* was prepared with the following formula: glucose, 30 g/L; yeast extract, 15 g/L; MgSO_4_·7H_2_O, 1 g/L; KH_2_PO_4_, 1.5 g/L; ZnSO_4_·7H_2_O, 0.06 g/L; pH 7.0. The medium was aliquoted into 1000 mL shake flasks, with 400 mL of medium per flask. Four uniform fungal plugs (0.5 cm^2^ each) were obtained using a sterile punch and inoculated into each flask. Cultivation was performed at 26 °C with agitation at 160 r/min for 10 days. After fermentation, the culture broth was filtered through a 60-mesh sieve, and the mycelium was rinsed with sterile water, collected, and dried in an oven at 50 °C to obtain *I. levis* mycelium.

A total of 300 g dried *I. levis* mycelium was weighed, ground, and sieved through an 80-mesh screen. The obtained powder was soaked in 4 volumes of absolute ethanol for 48 h, centrifuged at 12,000 rpm for 5 min, and the precipitate was collected and air-dried. Two liters of double-distilled water were added to the precipitate, and the mixture was extracted by boiling water bath for 1 h, followed by centrifugation at 12,000 rpm for 5 min to collect the supernatant. The extraction was repeated three times, and the combined supernatant was the crude extract. The crude extract was concentrated to 200 mL by rotary evaporation, 4 volumes of absolute ethanol were added, and the mixture was stored at 4 °C for 24 h. After centrifugation at 12,000 rpm for 5 min, the supernatant was collected and concentrated to 200 mL by rotary evaporation. This step was repeated five times to obtain the polysaccharide-free mycelium aqueous extract (15 g). The total sugar content of the aqueous extract was determined by the phenol–sulfuric acid method with glucose as the standard, and the residual polysaccharide content was less than 0.3% (*w*/*w*), confirming that the fraction was essentially polysaccharide-free.

The aqueous extract was further separated using a Sephadex LH-20 column (mobile phase: acetonitrile:water = 1:1) to obtain the active fraction WYS. The column specifications were 3 cm (outer diameter) × 30 cm (length) with a flow rate of 1 mL/min. The eluate was collected in 10 mL fractions and analyzed by thin-layer chromatography (TLC). Fractions with identical TLC profiles (same Rf values and similar color development) were combined, yielding 5 combined fractions in total. Based on preliminary MTT screening, the third fraction exhibited the strongest anti-proliferative activity against MCF-7 cells and was designated as WYS. The yield of WYS was approximately 1.85% (*w*/*w*) based on dry mycelium weight.

### 2.4. Chemical Composition Analysis of WYS

The active fraction WYS was analyzed using an ultra-performance liquid chromatograph coupled with a quadrupole time-of-flight mass spectrometer (UPLC-Q-TOF-MS, AB Sciex Exion-QTOF X500R, AB Sciex, Framingham, MA, USA). The chromatographic conditions were as follows: separation was performed on a Zorbax Eclipse C18 column (1.8 μm particle size, 2.1 mm × 100 mm, Agilent Technologies, Santa Clara, CA, USA) maintained at 30 °C, with a constant flow rate of 0.3 mL/min. The mobile phase comprised solvent A (0.1% formic acid aqueous solution) and solvent B (chromatographic-grade acetonitrile). WYS, solvent blank (pure water), and blank fermentation medium (processed through the same extraction and fractionation procedure) were injected and analyzed. Peaks present in blank samples were excluded from the final metabolite list to eliminate medium-derived and system background compounds. Three independent biological replicate samples of WYS were prepared, with two technical replicates per sample. Only metabolites detected in all biological replicates were included in the final list. The sample injection volume was fixed at 2 μL, and the autosampler chamber temperature was controlled at 4 °C. Both positive and negative electrospray ionization modes were adopted with consistent parameter settings: heater temperature, 325 °C; sheath gas flow, 45 arb; auxiliary gas flow, 15 arb; sweep gas flow, 1 arb; electrospray voltage, 3.5 kV; capillary temperature, 330 °C; and S-lens RF level, 55%. Data were collected in full scan mode (*m*/*z* 100–1500) coupled with data-dependent tandem mass spectrometry (dd-MS^2^, TopN = 5). The resolution was set at 120,000 for MS1 and 60,000 for MS2, and higher-energy collisional dissociation (HCD) was selected as the fragmentation mode. MS1 mass tolerance was set to 5 ppm; an MS/MS fragmentation matching score > 0.7 was considered a valid match. Metabolites were annotated by matching against HMDB, MassBank, MetaboAnalyst, and PubChem databases. All annotations correspond to MSI Level 2.

### 2.5. Identification of Targets of WYS Against Breast Cancer

Compounds with a relative abundance greater than 0.1% in the full-spectrum data of positive and negative ion modes were selected as key metabolites. A total of 72 key metabolites were screened in the positive ion mode and 89 in the negative ion mode. After merging and deduplication (metabolites with identical compound names and molecular formulas were considered the same substance; for compounds detected in both modes, the one with a higher MS/MS matching score was retained), 152 key metabolites were identified for subsequent analysis. The chemical structures and molecular information of the key metabolites were obtained from the PubChem database [22], and the target genes of the key metabolites were predicted using the Swiss Target Prediction database with a probability threshold > 0.1, with only human-derived targets retained. Using “Breast cancer” as the keyword, potential disease-related targets were mined from the OMIM database (https://omim.org/ (accessed on 2 January 2026)) and the GeneCards database (http://www.genecards.org/ (accessed on 2 January 2026)) (union of multiple databases) to obtain breast cancer disease-related genes. The R software (version 4.4.1), with package DESeq2 (version 1.38.3, https://bioconductor.org/packages//2.13/bioc/html/DESeq2.html (accessed on 2 January 2026)) was used for differential expression analysis of the training set TCGA-BRCA (BRCA vs. Control). The differential screening criteria were *p* value < 0.05 and |log2FC (fold change)| > 1.5, to screen for significantly differentially expressed genes (DEGs) between breast cancer samples and control samples. Finally, intersection analysis was performed on the key metabolite target genes, disease target genes and differentially expressed genes to obtain differentially expressed common targets.

### 2.6. GO and KEGG Enrichment Analysis

To explore the biological processes and signaling pathways involved in the differentially expressed common targets, the R package “clusterProfiler” (version 4.14.6, https://bioconductor.org/packages/release/bioc/html/clusterProfiler.htm (accessed on 2 January 2026)) was used for Gene Ontology (GO) enrichment and Kyoto Encyclopedia of Genes and Genomes (KEGG) pathway enrichment analyses of the genes, aiming to explore their biological pathways [23]. GO analysis describes the potential functions and pathways of candidate genes from three aspects: biological process (BPs), molecular function (MF), and cellular component (CC). KEGG analysis helps identify the metabolic and signaling pathways involving the genes, providing an understanding of gene function. Visualization was performed using the R package “ggplot2” (version 3.5.2, https://ggplot2.tidyverse.org/).

### 2.7. PPI Network Construction and Candidate Gene Acquisition

To explore the protein–protein interactions encoded by the differentially expressed common targets, the obtained differentially expressed common targets were submitted into the STRING database (https://string-db.org/). Species was defined as Homo sapiens, and the interaction threshold was set at 0.4 to build the protein–protein interaction (PPI) network [24]. The PPI network obtained from the STRING database was imported into Cytoscape software (version 3.9.1, https://cytoscape.org) for network visualization and topological analysis [25].

Subsequently, the CytoHubba plugin (http://apps.cytoscape.org/apps/CytoHubba (accessed on 2 January 2026)) in Cytoscape software was used to evaluate the topological characteristics of network nodes (candidate genes), including Maximum Clique Centrality (MCC), Maximum Neighborhood Component (MNC), Edge Percolation Component (EPC) and Degree. The top 40 genes ranked by each topological parameter were selected, and the intersection of the results from the four methods was taken to obtain the intersection genes, designated as candidate genes for subsequent analysis.

### 2.8. Screening of Characteristic Genes Using Machine Learning Algorithms

After obtaining the candidate genes, LASSO, Random Forest, and SVM-RFE were constructed using the R packages glmnet (version 4.1.10, https://github.com/cran/glmnet (accessed on 3 January 2026)), caret (version 7.0.1, https://cran.r-project.org/web/packages/caret/index.html (accessed on 3 January 2026)), and randomForest (version 4.7-1, https://cran.r-project.org/src/contrib/Archive/randomForest/ (accessed on 3 January 2026)), respectively, with the diagnostic result as the target vector, to screen key genes [26]. All three models adopted 10-fold cross-validation during training: LASSO selected the optimal lambda value via cross-validation; SVM-RFE determined the optimal feature number based on 10-fold cross-validation error; Random Forest evaluated feature importance based on out-of-bag (OOB) error. The genes screened by the three algorithms were intersected, and the intersection genes were regarded as disease characteristic genes. The intersection strategy was adopted to integrate the advantages of algorithms with different principles, reduce the bias of a single method, and improve the robustness and reliability of the screened characteristic genes.

### 2.9. Expression and ROC Validation of Characteristic Genes

After obtaining the characteristic genes, their expression values were extracted from the training set TCGA-BRCA and the validation set GSE10810. The Wilcoxon test was used to calculate the expression differences between disease and control groups, and boxplots were drawn using the R package “ggplot2”. Genes with consistent expression trends (upregulated or downregulated in the disease group compared with the control group) and significant differences (*p* < 0.05) in both the training and validation sets were retained. Then, the R package pROC (version 1.18.5, https://cran.r-project.org/src/contrib/Archive/pROC/ (accessed on 4 January 2026)) was used to calculate the Area Under the Curve (AUC) values of these genes for sample outcome prediction, and ROC curves were plotted [27]. Genes with AUC > 0.7 in both datasets were defined as final characteristic genes. Meanwhile, integrating the expression of characteristic genes, Spearman correlation analysis was performed using the R package “PerformanceAnalytics” (version 2.0.8, https://CRAN.R-project.org/package=PerformanceAnalytics (accessed on 4 January 2026)), and correlation scatter trend plots were drawn.

### 2.10. Construction of Active Metabolite–Characteristic Gene Network

Characteristic genes and their corresponding key metabolites were screened, and pathways enriched with at least 4 characteristic genes in the single-gene GSEA were selected. Cytoscape software was used to construct a disease-active metabolite-characteristic gene-KEGG pathway network. Then, the chemical structures and molecular information of the corresponding metabolites were obtained from the PubChem database (https://pubchem.ncbi.nlm.nih.gov (accessed on 4 January 2026)), and the physicochemical properties of the key metabolites were predicted using the ADMETLAB 3.0 platform (https://admetlab3.scbdd.com).

### 2.11. Molecular Docking

The SDF structural information of metabolites corresponding to characteristic genes was obtained from the PubChem database (https://pubchem.ncbi.nlm.nih.gov), and the 3D structures of proteins corresponding to characteristic genes were downloaded from the Protein Data Bank (PDB) (https://www.rcsb.org/); PDB accession numbers: EGFR (1M17), CDK1 (4Y72), TOP2A (1ZXM), KIT (1T46), and FGF2 (1BFC). Pymol software (version 2.6.0a0 Open-Source, http://www.pymol.org) was used to remove water molecules and original ligands from the protein complexes, retaining only the monomeric structure of the protein corresponding to the characteristic gene. The processed protein was imported into AutoDock Tools (version 1.5.7, https://ccsb.scripps.edu/ (accessed on 4 January 2026)) for hydrogenation, charge calculation, and non-polar hydrogen combination [28]. Subsequently, docking parameters were set as follows: exhaustiveness = 20, number of modes = 9. Grid box center and size were individually adjusted for each protein to cover the entire active pocket, molecular docking was performed, and the results were visualized using Pymol software (version 2.6.0a0, open-source, http://www.pymol.org).

### 2.12. Molecular Dynamics Simulation

To verify the molecular docking results of characteristic genes and key metabolites, all-atom molecular dynamics simulations were carried out with Gromacs 2021.4 software. The small-molecule topology file was constructed via the sobtop tool, while protein structural and topological files were processed with native Gromacs utilities. The Amber14SB force field was used for proteins and the GAFF force field for small molecules, with the SPC water model. To simulate the physiological environment, the system was neutralized with Na^+^/Cl^−^ ions. Molecular mechanics optimization was performed using the steepest descent method, followed by NVT ensemble equilibration at 300 K for 100 ps and NPT ensemble equilibration at 1 bar for 100 ps under constrained system position. After equilibration, a 100 ns molecular dynamics simulation was conducted. Key indicators such as the Root Mean Square Deviation (RMSD) of the protein–small-molecule complex and the radius of gyration of the protein were calculated, Gibbs free energy landscape maps were drawn, and the simulation results were visualized using DuIvytools software (version 0.6.0, https://duivytools.readthedocs.io/en/latest/DIT.html (accessed on 4 January 2026)). To ensure the reproducibility of the simulation results, two independent 100 ns production trajectories were generated for each complex starting from different initial velocity distributions. Consistency between replicates was evaluated by comparing the convergence trends of RMSD, RMSF, and Rg values. Both independent trajectories exhibited highly similar equilibrium profiles and consistent stability conclusions.

### 2.13. Cell Culture

MCF-7 cells were maintained in RPMI-1640 complete medium containing 10% fetal bovine serum and antibiotic mixture (100 μg/mL penicillin plus 100 μg/mL streptomycin). Incubation conditions were set as 37 °C with 5% CO_2_ in a humidified chamber. Cells were passaged every 48 h at a ratio of 1:3 using trypsin. All cellular experiments included at least 3 independent biological replicates, with 3 technical replicates per sample.

### 2.14. Cell Proliferation Inhibition Assay

A volume of 100 μL cell suspension (1 × 10^4^ cells/well) was plated in 96-well plates and cultured for 24 h under 37 °C and 5% CO_2_. WYS aqueous solution at concentrations of 3.75, 7.5, 15, 30, 60, 120, and 240 μg/mL was added to each well, with an equal volume of sterile water as the control. After 24, 36, and 48 h of treatment, morphological changes were observed under an inverted microscope, and the inhibitory rate of WYS on MCF-7 cells was determined via the MTT assay. The half-maximal inhibitory concentration (IC_50_) of the active fraction was calculated using SPSS 13.0 software [29].

### 2.15. Cell Apoptosis Assay

Cells were plated in 6-well plates at 2 × 10^5^ cells/well and cultured for 24 h to achieve full attachment. Subsequently, WYS aqueous solutions were supplemented to reach final concentrations of 15, 30, and 60 μg/mL in individual wells; equivalent sterile water was set as the blank control, and cells were further incubated for 48 h. After digestion using 0.25% trypsin, the cells were harvested via centrifugation, rinsed twice with ice-cold PBS at 4 °C, and reconstituted in 500 μL of binding buffer to obtain a cell suspension of 1 × 10^7^ cells/mL. A total of 100 μL cell suspension was pipetted into a 5 mL flow tube, blended with 5 μL Annexin V-FITC, and supplemented with 5 μL PI for staining. The staining system was kept at ambient temperature under dark conditions for 15 min before flow cytometric detection. The intact cell population was first gated by FSC/SSC to exclude debris, then single cells were selected via FSC-A/FSC-H doublet exclusion, and finally divided into four quadrants based on FITC and PI fluorescence intensities for apoptosis quantification.

### 2.16. Immunofluorescence Staining

Cells were seeded into 6-well plates with sterile coverslips at a density of 2 × 10^5^ cells/well and cultured at 37 °C with 5% CO_2_ until reaching 75% confluence. Subsequently, cells were treated with sterile water (control group) and different concentrations of WYS (designated WYS-15, WYS-30, and WYS-60) for 48 h. For mitochondrial labeling, the cells were incubated with pre-warmed MitoTracker Red CMXRos working solution (final concentration 200 nM) in serum-free medium at 37 °C in the dark for 20 min. After washing three times with pre-warmed PBS, the cells were immobilized using 4% paraformaldehyde for 15 min at ambient temperature, followed by permeabilization with 0.1% Triton X-100 for 5 min and counterstaining with DAPI for 10 min to visualize the nuclei. Coverslips were mounted with anti-fade mounting medium, and images were acquired using a laser scanning confocal microscope (scale bar = 50 μm) with consistent acquisition parameters across groups. Blue (DAPI) and red (MitoTracker) fluorescence channel signals were sequentially collected, and merged images were generated for colocalization analysis.

### 2.17. Cell Cycle Arrest Assay

Flow cytometry was used to detect the effect of WYS on the cell cycle of MCF-7 cells. WYS was set at three concentrations (15, 30 and 60 μg/mL) with sterile water as the control. After 48 h of treatment, cells were digested, collected by centrifugation, washed with normal saline, counted and adjusted to a concentration of 1 × 10^6^ cells/mL. Cells were fixed overnight with 70% ice-cold ethanol, and DNA fluorescence staining was performed using propidium iodide (PI). One milliliter of PI staining solution (50 μg/mL PI, 10 μg/mL RNase and 1% Triton X-100) was added to each sample, which was then stained at 4 °C for 30 min and analyzed using a flow cytometer for cell cycle distribution.

### 2.18. Mitochondrial Membrane Potential Assay

JC-1, enables rapid detection of ΔΨm depolarization via changes in its red-to-green fluorescence ratio, which acts as an early indicator of cell apoptosis. [30]. Cells were cultured in 6-well plates (2 × 10^5^ cells/well) for 12 h, then treated with WYS at gradient concentrations (15, 30 and 60 μg/mL) and sterile water (negative control group) for 48 h. Cells were collected and resuspended in 0.5 mL cell culture medium at a concentration of 2 × 10^5^ cells/mL. Half a milliliter of JC-1 staining working solution was added, mixed by inversion, and incubated at 37 °C in a cell incubator for 20 min. Cells were collected by centrifugation at 600 g for 3–4 min at 4 °C, washed twice with JC-1 staining buffer (PBS), and analyzed using a flow cytometer.

### 2.19. Western Blot Analysis

MCF-7 cells were exposed to WYS aqueous solution at serial concentrations (15, 30 and 60 μg/mL), while sterile water served as the negative control for a 48 h treatment period. Cells were lysed on ice for 30 min with RIPA buffer supplemented with protease and phosphatase inhibitors. The lysate was centrifuged at 12,000× *g* and 4 °C for 15 min to harvest the supernatant. A BCA assay kit was applied to measure total protein concentration. Identical protein loads (30 μg per lane) were resolved via 8–12% SDS-PAGE and electrotransferred onto PVDF membranes. The membranes were blocked with 5% skim milk or 5% BSA solution at ambient temperature for 1 h, then incubated with specific primary antibodies (cleaved caspase-3, cleaved caspase-9, cleaved PARP, P-EGFR, P-MEK, P-ERK1/2, P53, cytochrome C, Bcl-2, Bax, and so on) overnight at 4 °C. Following three rounds of 10 min TBST rinsing, the membranes were probed with horseradish peroxidase-conjugated secondary antibodies for 1 h at room temperature. Protein bands were visualized using an ECL chemiluminescence kit and captured with a chemiluminescence imager. ImageJ software (version 1.8.0) was used to analyze band grayscale intensity. For total proteins, band intensity was normalized to the internal reference GAPDH. For phosphorylated proteins, band intensity was first normalized to GAPDH, and then the ratio of phosphorylated protein to corresponding total protein was calculated to represent the relative phosphorylation level.

### 2.20. In Vivo Mouse Experiment

Four-week-old female BALB/c nude mice of specific pathogen-free (SPF) grade were purchased from Hunan Slac Jingda Laboratory Animal Co., Ltd., Changsha, China (Laboratory Animal Production License No. SCXK (Xiang) 2019-0004.) All animals were housed in the SPF-grade laboratory animal center of Hunan Institute of Microbiology (Changsha, China) at a controlled temperature of 22–24 °C with 50–60% relative humidity and a 12 h light/12 h dark cycle. The mice had free access to sterilized standard chow and sterile drinking water, and were acclimatized for one week prior to the experiment. Mice with palpable hard tumor nodules (approximately 100 mm^3^) at 7 days after subcutaneous inoculation were enrolled in the study. The animals were allocated to 5 groups (6 mice per group) using a random-number-table method: blank control group (equal volume of sterile normal saline, intragastric administration), WYS low-dose group (40 mg/kg, intragastric administration), WYS medium-dose group (80 mg/kg, intragastric administration), WYS high-dose group (160 mg/kg, intragastric administration), and cisplatin (CDDP)-positive control group (3 mg/kg, intraperitoneal injection). Drugs were administered once every 3 days, with a dosing volume of 0.2 mL per mouse. Pre-specified inclusion and exclusion criteria are as follows. Inclusion criteria: mice with successful tumor formation and good mental status after inoculation; exclusion criteria: mice with failed tumor formation, severe infection, or accidental death. No animals were excluded from this experiment. Humane endpoints: the general state, activity, and food intake of the animals were monitored daily. Body weight was recorded every 3 days as an indicator of treatment tolerability. Euthanasia by cervical dislocation was performed when the tumor volume in the control group reached 1100 mm^3^, or when animals showed severe distress, such as tumor ulceration, body weight loss exceeding 20%, or inability to eat and drink. All animals in this study were sacrificed uniformly when the tumor volume of the control group reached the pre-specified endpoint. The longest diameter (L) and shortest diameter (W) of tumors were measured using a vernier caliper, and tumor volume was calculated using the formula V = 0.5 × L × W^2^.

### 2.21. Tissue Sectioning and Staining

Excised mouse tumor tissues were fixed, paraffin-embedded, and cut into 5 μm thick slices. Following deparaffinization and serial ethanol rehydration, tissue slices were permeabilized with 20 μg/mL proteinase K for 15 min under ambient temperature. Apoptotic cells were then visualized via a one-step TUNEL staining kit. The sections were reacted with TUNEL working solution at 37 °C in darkness for 60 min, and DAPI staining was applied for 5 min to label nuclei. Micrographs were captured with a laser scanning confocal microscope. Five randomly selected visual fields from each group were analyzed to calculate the percentage of TUNEL-positive cells for evaluating cellular apoptosis.

### 2.22. Statistical Analysis

All experiments were independently repeated at least three times, and data were expressed as mean ± standard deviation (mean ± SD). Statistical analysis was performed using GraphPad Prism 9.0 software. Comparisons between two groups were conducted using an independent-samples *t*-test. For longitudinal xenograft tumor volume data, a repeated-measures ANOVA (RM-ANOVA) with Greenhouse–Geisser sphericity correction was used, followed by Bonferroni post hoc tests. Partial eta-squared was reported as an effect size, and 95% confidence intervals of mean differences were provided for key comparisons. *p* < 0.05 was considered statistically significant.

## 3. Results

### 3.1. Potential Targets of WYS in the Treatment of Breast Cancer

**Figure 1:**
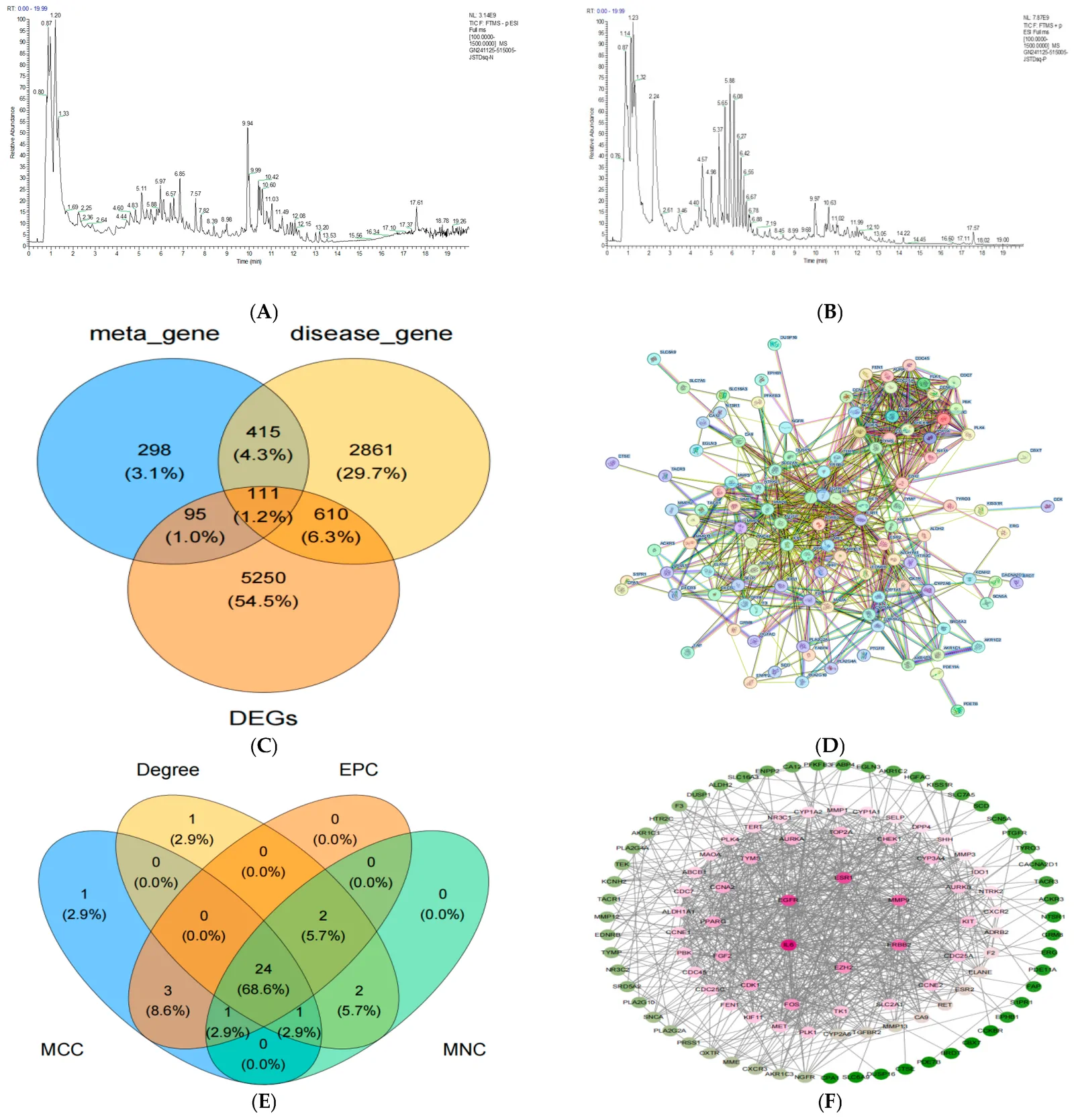
Identification of key targets for WYS in the treatment of breast cancer using network pharmacology. (**A**) MS spectrum of WYS in positive ion mode. (**B**) MS spectrum of WYS in negative ion mode. (**C**) Venn diagram of potential therapeutic targets. The blue circles represent key metabolite target genes, the yellow circles represent disease-related genes, and the orange circles represent DEGs. The overlapping region indicates differentially expressed common targets. (**D**) The PPI network generated by the STRING database. (**E**) Venn diagram for candidate gene screening. (**F**) The PPI network constructed using Cytoscape software. The nodes represent potential target genes; node colors are adjusted according to degree values, with higher values in pink and lower values in green.

Compounds with a relative peak area greater than 0.1% (semi-quantitative estimation based on total ion current) in the full-spectrum data under positive and negative ion modes were selected as key metabolites. The 0.1% relative abundance threshold is a commonly used cutoff in untargeted metabolomics studies. It can effectively eliminate low-signal peaks with poor repeatability and high background noise, reduce false positive annotations, and ensure the reliability of subsequent target prediction. Among them, 72 key metabolites were screened under positive ion mode (Figure 1A, Supplementary Table S1), and 89 key metabolites were screened under negative ion mode (Figure 1B, Supplementary Table S2). After merging and deduplication, 152 key metabolites were identified for subsequent analysis. Next, the Swiss Target Prediction database was used to predict metabolite target genes, and 919 metabolite target genes were obtained after merging and deduplication. With “Breast cancer” as the keyword, 3897 disease-related target genes were predicted from the GeneCards database, and 196 disease-related target genes were predicted from the OMIM database. The union of disease-related target genes predicted by each database was taken and deduplicated to obtain 3997 disease-related target genes. Next, the intersection of 919 key metabolite target genes, 3997 disease-related target genes, and 3539 DEGs was taken, and, finally, 111 differential common targets were obtained (Figure 1C).

### 3.2. PPI Network Construction and Candidate Key Gene Acquisition

To explore the synergistic regulatory relationship between common targets of disease and drugs, the STRING database was used to construct a protein–protein interaction (PPI) network for these 111 differential common targets (Figure 1D). Network nodes represent core target genes; the node connections represent functional interactions between proteins; and the density of connections reflects the strength of protein binding and the degree of pathway synergy. The results were imported into Cytoscape software to visualize the obtained PPI network (Figure 1F). After deleting the isolated nodes, the network diagram included 80 nodes and 442 edges. The results showed that there was a close and orderly regulatory relationship between anti-breast cancer targets, highlighting the multi-target synergistic regulation mode of *I. levis*. Then, four methods (MCC, Degree, MNC, and EPC) in Cytohubba were used to analyze the topological properties of the PPI network, calculate the importance score of each differential common target, and rank the genes according to the score. The blue circle represents the top 30 genes with the highest importance score obtained by the MCC method; the orange circle represents the top 30 genes with the highest importance score obtained by the EPC method; the yellow circle represents the top 30 genes with the highest importance score obtained by the Degree method; and the green circle represents the top 30 genes with the highest importance score obtained by the MNC method. The overlapping part in the middle is the intersection gene, defined as the candidate gene. The intersection of the top 40 genes ranked by the four methods was taken to obtain key genes. Finally, 24 candidate genes were screened (Figure 1E).

### 3.3. Enrichment Analysis

**Figure 2:**
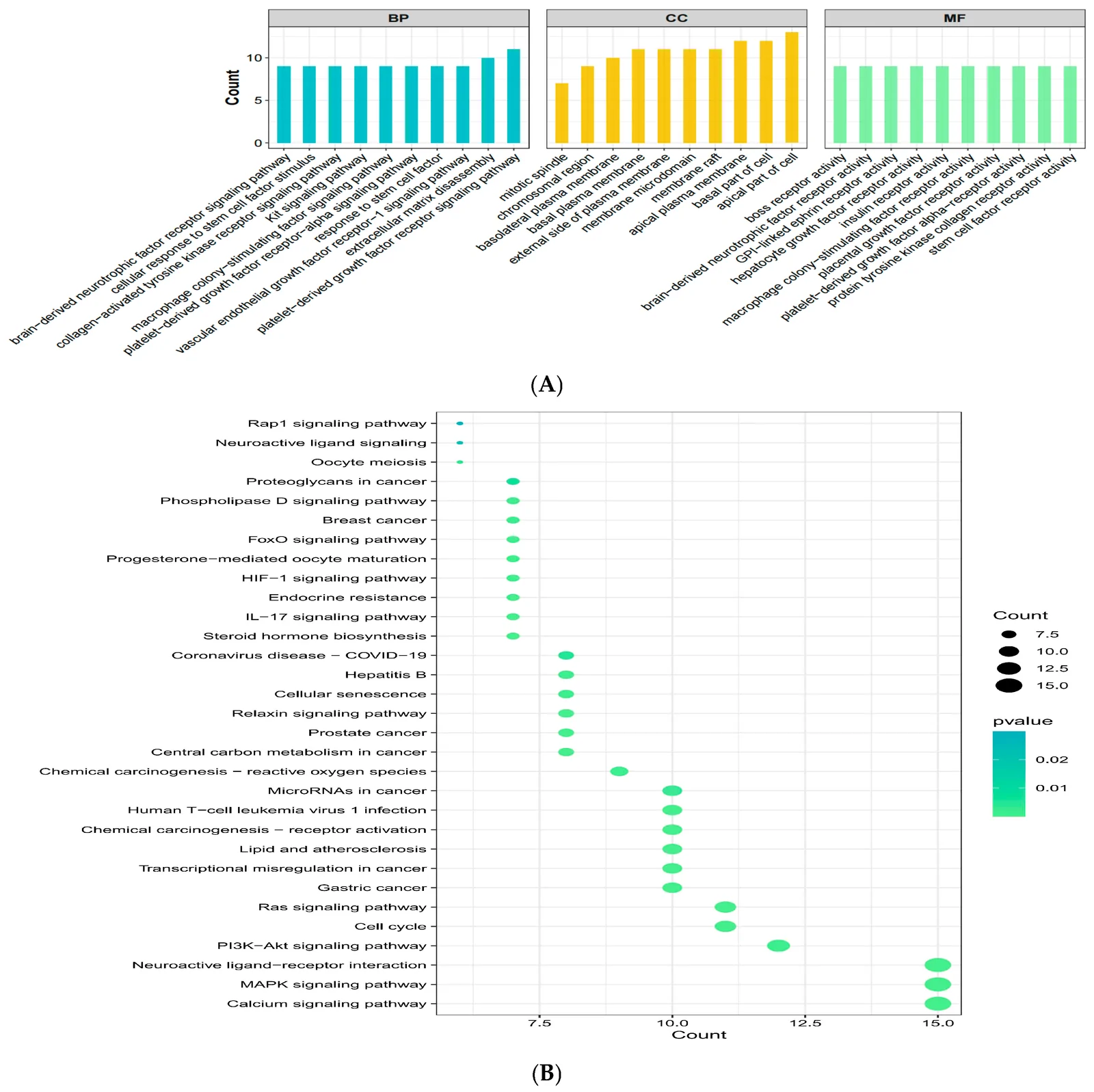
GO and KEGG enrichment analyses of differentially expressed common targets. (**A**) Bar plot of GO enrichment analysis. Different panels represent BP, CC, and MF, respectively. The vertical axis represents the number of differentially expressed common genes enriched in each term. (**B**) Bubble plot of KEGG enrichment analysis. The horizontal axis represents the number of genes enriched in each pathway. The vertical axis represents KEGG pathways. The bubble size indicates the number of enriched genes, and the bubble color indicates the *p*-value.

According to the above method, the R package clusterProfiler was used to perform GO and KEGG enrichment analysis on 111 differential common targets, with a significance threshold of *p*-value < 0.05 and count > 5. A total of 439 GO enrichment pathways (367 BP, 15 CC, and 57 MF) and 31 KEGG pathways were enriched. The top five GO pathways (BP/CC/MF) and all KEGG pathways were selected for visualization according to *p*-value sorting. In the GO pathways (Figure 2A), for BP, differential genes were highly involved in growth factor receptor signaling, stem cell response and immune/vascular-related signaling pathways. For CC, the corresponding gene products were mainly located in cell membrane microdomains, plasma membrane, and cell division-related structures (spindle, chromosome). For MF, the core focus was on receptor tyrosine kinase activity, especially the functions of various growth factor and cytokine receptors. In KEGG, differential genes were significantly concentrated in the MAPK and PI3K-Akt pathways. At the same time, they were highly associated with tumor progression, endocrine resistance, and inflammation/aging microenvironment-related pathways (Figure 2B). Therefore, the GO functional classification and KEGG pathway enrichment were highly consistent. These differential genes centrally regulated cell proliferation, cycle, and survival through the mechanism of membrane receptor tyrosine kinase activation and downstream oncogenic signaling pathway crosstalk, providing a clear functional and pathway basis for subsequent mechanism verification and targeted intervention.

### 3.4. Machine Learning for Screening Feature Genes

**Figure 3:**
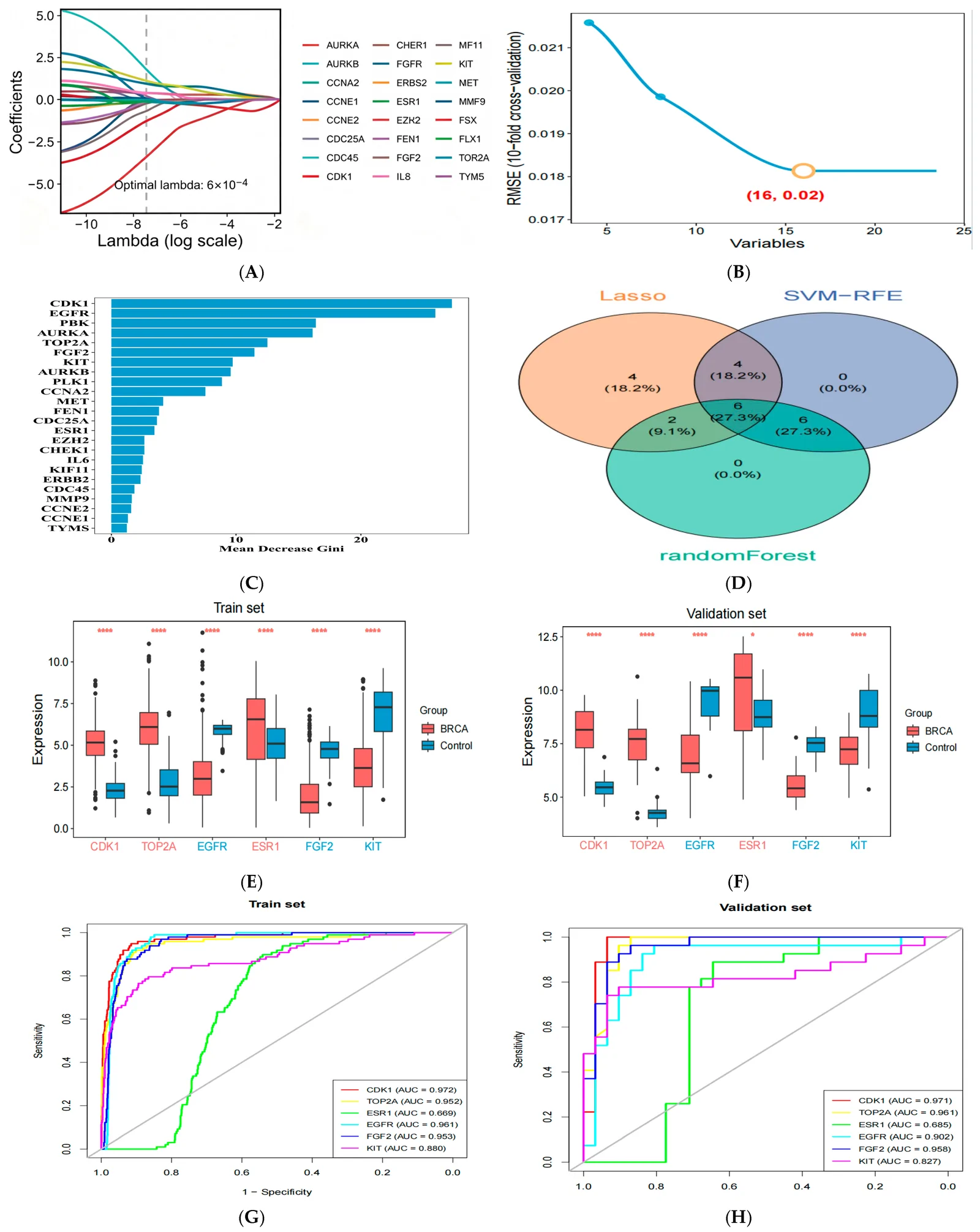
Screening, expression analysis, and ROC validation of feature genes. (**A**) LASSO regression for feature gene selection. The horizontal axis represents the logarithmic value of Lambda, and the vertical axis represents the coefficient estimates of each variable in the LASSO regression. (**B**) SVM-RFE for feature gene selection. The horizontal axis represents the number of variables included in the model; the vertical axis represents the root mean square error of 10-fold cross-validation, an indicator of model prediction error, where a smaller value indicates a lower error. The blue dots represent RMSE values obtained from 10-fold cross-validation under different numbers of variables. The orange hollow circle marks the optimal point with 16 variables and cross-validation RMSE of 0.02. (**C**) Random Forest for feature gene selection. The horizontal axis represents the importance score, and the vertical axis represents genes, showing the importance ranking of feature genes. (**D**) Venn diagram of overlapping feature genes. The orange circle represents genes screened by LASSO, the green circle represents genes screened by Random Forest, and the purple circle represents genes screened by SVM-RFE. The overlapping regions indicate the intersection genes of the three machine learning algorithms. (**E**,**F**) Boxplots of feature gene expression in the training and validation sets. The horizontal axis represents gene names, and the vertical axis represents the distribution of gene expression levels. Different colors represent groups: orange for the disease group and blue for the control group. Statistical significance is marked with * (*p* < 0.05), **** (*p* < 0.0001), and ns indicates no significant difference. (**G**) ROC curves of feature genes in the training set. (**H**) ROC curves of feature genes in the validation set.

Machine learning analysis was performed using the TCGA-BRCA training set to further screen 24 candidate genes. The LASSO results are shown in Figure 3A; 16 feature genes were obtained. The SVM-RFE results are shown in Figure 3B. When the accuracy was the highest and the error rate was the lowest, 16 feature genes were screened. The RF feature importance results are shown in Figure 3C. According to the importance score sorting, genes with importance score greater than 3 were regarded as important genes of RF, and 14 feature genes were finally screened. Then, the intersection of these feature genes was taken, as shown in Figure 3D, and six feature genes were obtained, namely CDK1, TOP2A, EGFR, ESR1, FGF2, and KIT.

### 3.5. Expression and ROC Validation of Feature Genes

The six feature genes obtained above were tested in the TCGA-BRCA training set and the GSE10810 validation set, respectively. The differential box plots of feature genes in the training set and validation set are shown in Figure 3E,F. Among them, six genes (EGFR, CDK1, TOP2A, FGF2, KIT, and ESR1) had consistent expression trends and statistically significant differences (*p* < 0.05) in both the training set and validation set.

Next, ROC analysis was performed on the six genes and ROC curves were drawn. Genes with AUC > 0.7 in both the training set and validation set were selected as final feature genes. ESR1 was excluded because its AUC was 0.669 in the training set and 0.685 in the validation set, both below the 0.7 threshold. Finally, five feature genes (EGFR, CDK1, TOP2A, FGF2, and KIT) were retained (Figure 3G,H).

### 3.6. Disease–Active Metabolite–Feature Gene–KEGG Pathway Network Construction

**Figure 4:**
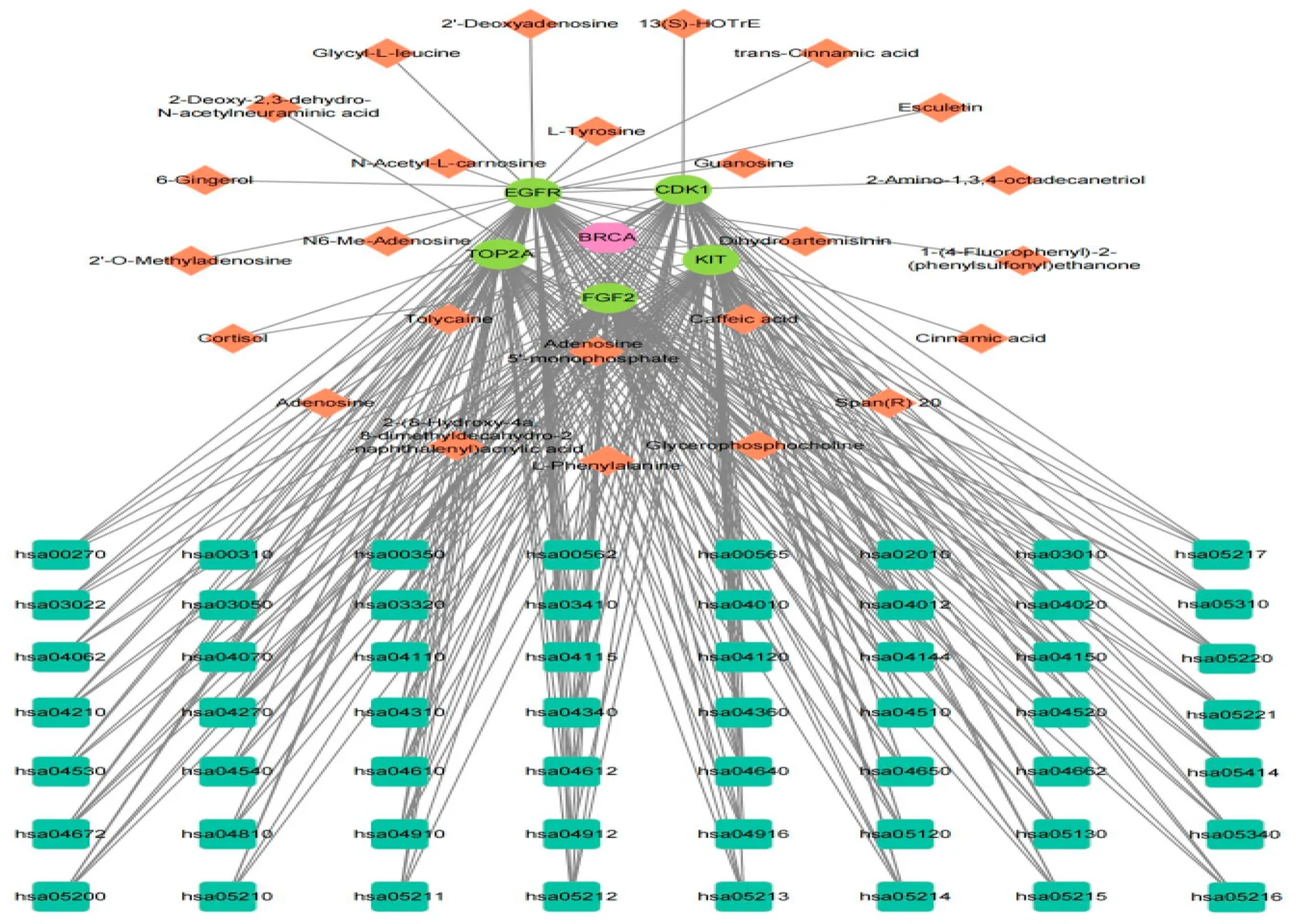
Disease–active metabolite–feature gene–KEGG pathway network. The pink nodes represent the BRCA disease; the inner light green nodes represent the feature genes; the outer dark green square nodes represent the metabolites; the green square nodes represent the KEGG IDs of pathways enriched in feature genes.

GSEA analysis was performed on the five feature genes, respectively, to obtain feature gene-enriched pathways (Supplementary Figure S1). Key metabolites corresponding to feature genes and KEGG pathways corresponding to feature genes were screened. Cytoscape 3.9.1 software was used to construct and visualize the “disease-metabolite-pathway-target” regulatory network (Figure 4). The network included five disease-related core targets (light green circular nodes), 25 metabolite active components (orange diamond nodes), and 56 core KEGG pathways (square green nodes, screened from GSEA enrichment results with ≥4 feature genes enriched). The table shows the metabolites corresponding to feature genes, including important compounds, such as dihydroartemisinin, guanosine, cinnamic acid, and cortisol, and suggests important signaling pathways: cell cycle arrest, mitochondrial apoptosis activation, MAPK pathway inhibition.

### 3.7. Molecular Docking Analysis

**Figure 5:**
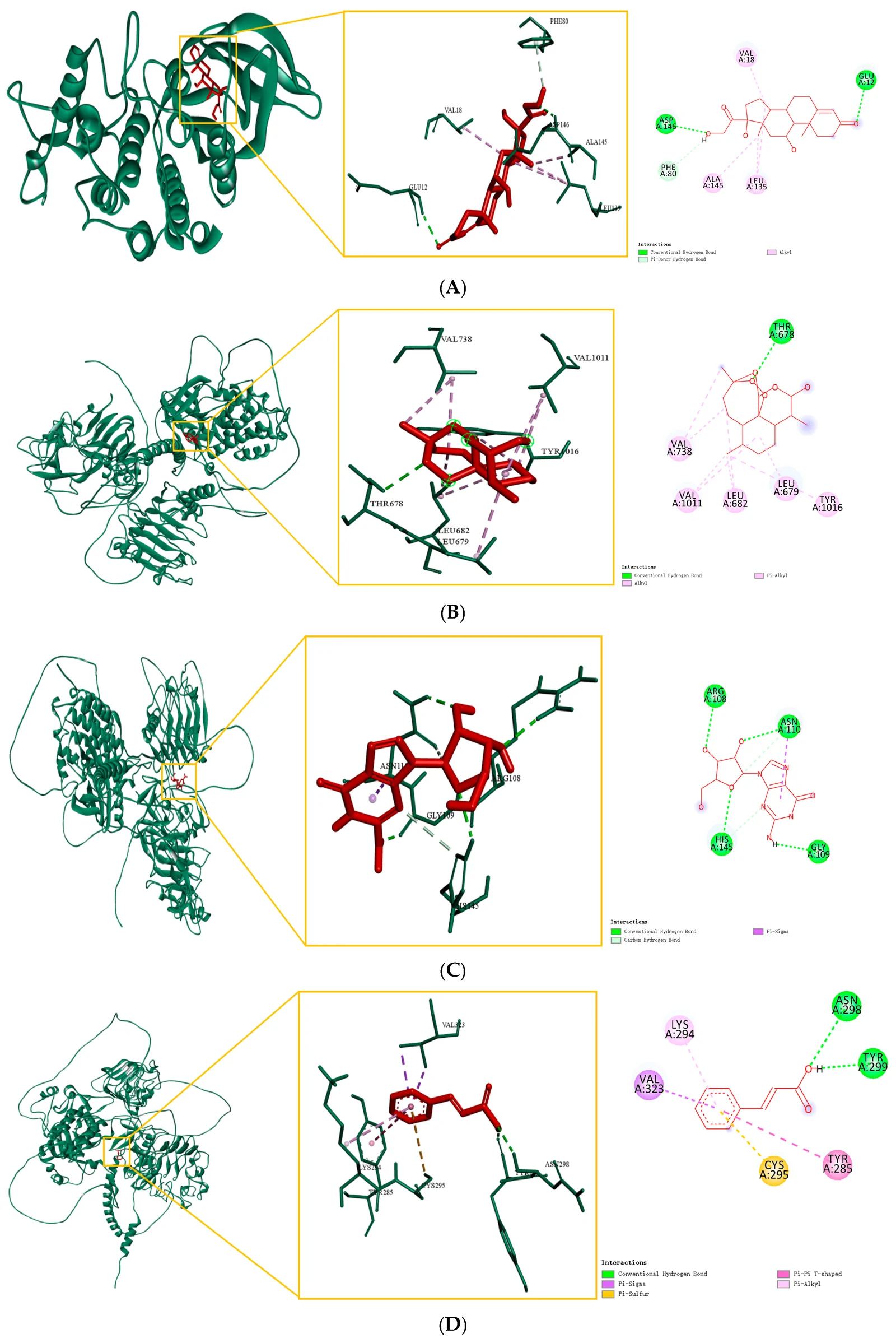

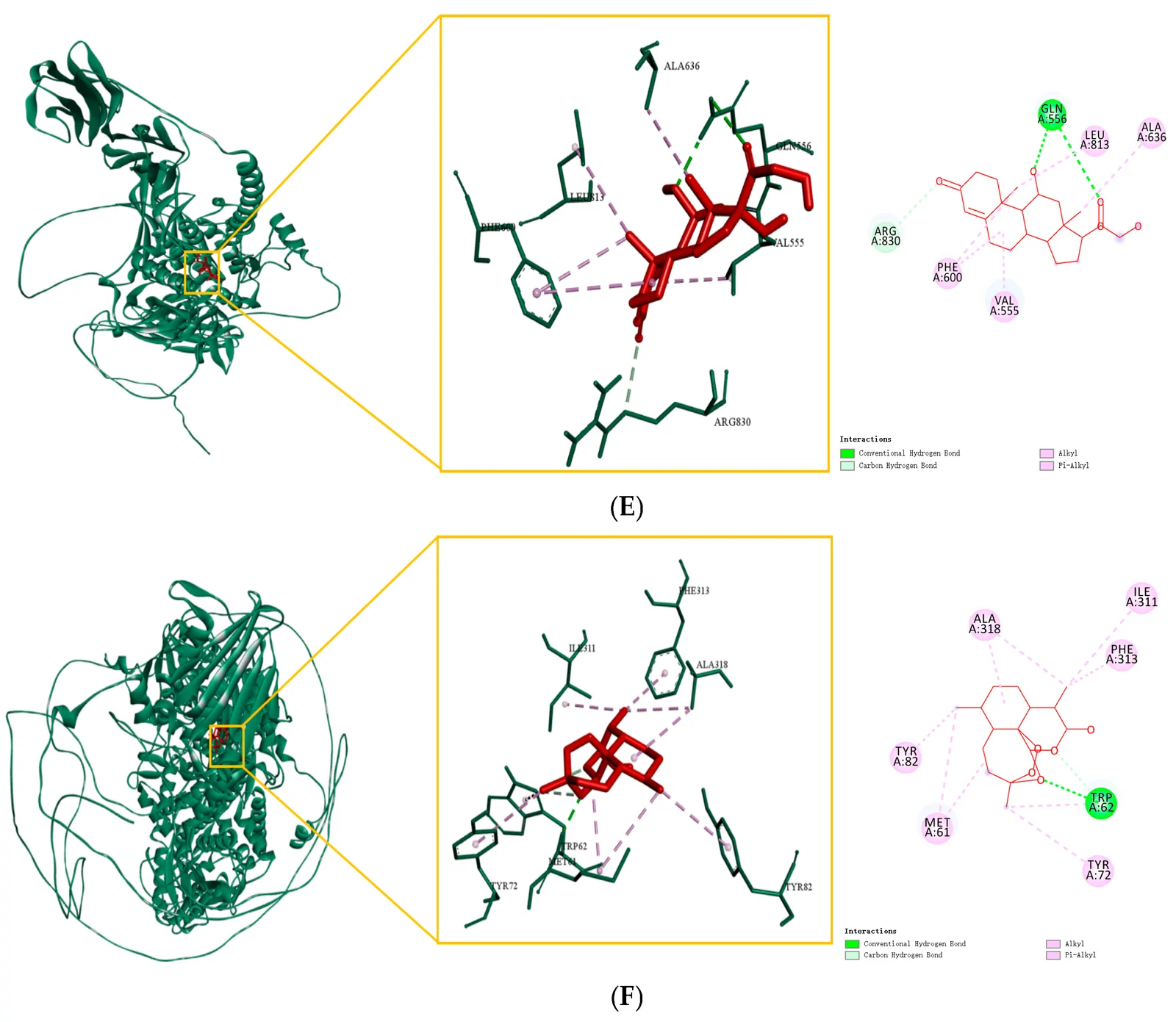
Molecular docking and interaction analyses between key metabolites and target proteins (3D and 2D structural diagrams). (**A**–**F**) Molecular docking of complexes: CDK1–cortisol, EGFR–dihydroartemisinin, EGFR–guanosine, EGFR–cinnamic acid, KIT–cortisol, and TOP2A–dihydroartemisinin.

To verify the potential binding ability of feature genes and active metabolites, molecular docking analysis was carried out. The results showed that all five key feature genes could spontaneously form stable complexes with the corresponding active metabolites; the binding energies were all negative; and the affinity levels were different. Among them, CDK1 had the strongest binding force with cortisol, with a binding energy of −8.104 kcal/mol; the binding energies of KIT with cortisol, TOP2A with dihydroartemisinin, EGFR with dihydroartemisinin, EGFR with guanosine, and EGFR with cinnamic acid were −7.503 kcal/mol, −7.343 kcal/mol, −7.214 kcal/mol, −7.209 kcal/mol, and −5.474 kcal/mol, respectively, all indicating favorable predicted binding affinities (Table 1). The docking visualization results further revealed the binding mode of each system. All metabolites could be embedded into the active pocket of the target protein, forming a stable binding network with key amino acid residues through various intermolecular forces, such as hydrogen bonds and hydrophobic interactions (Figure 5). The molecular docking results indicate the potential binding capacity between feature genes and active metabolites at the structural level, providing an important structural biology basis for subsequent in-depth exploration of the molecular mechanism regulating breast cancer progression.

### 3.8. Molecular Dynamics Simulation Analysis

After molecular docking, the two ligand–protein complexes with the lowest binding affinity (EGFR complexes with dihydroartemisinin and cinnamic acid, respectively) were selected for validation by 100 ns molecular dynamics simulations. Three key indicators, RMSD, RMSF, and the radius of gyration (Rg), were used to systematically evaluate the structural stability and conformational dynamic changes of the complexes during the simulation.

**Figure 6:**
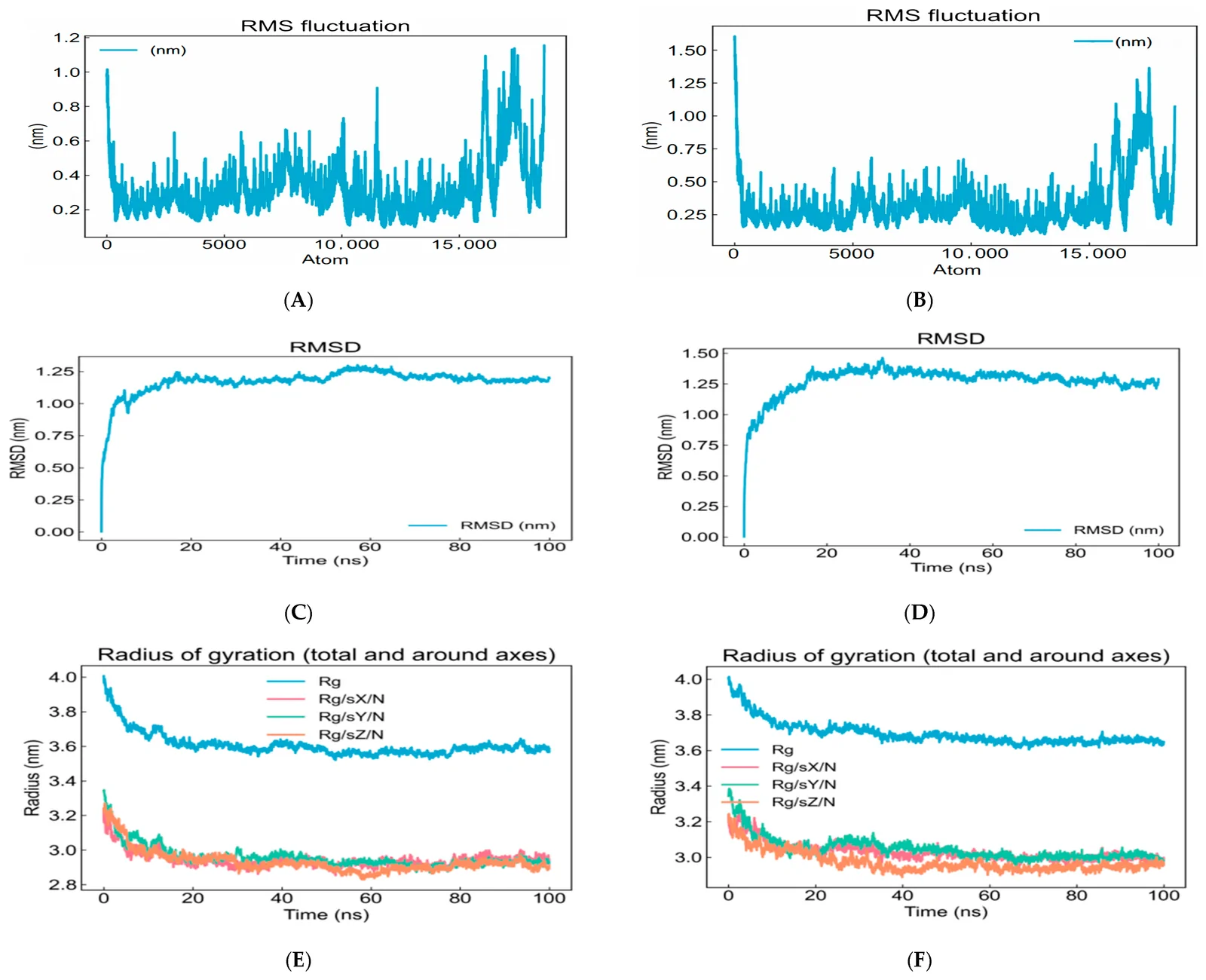
Molecular dynamics simulation validation of the stability of core compounds and their target proteins. (**A**,**C**,**E**) RMSF, RMSD, and Rg analyses of the EGFR–dihydroartemisinin complex during molecular dynamics simulation. (**B**,**D**,**F**) RMSF, RMSD, and Rg analyses of the EGFR–-cinnamic acid complex during molecular dynamics simulation.

For the EGFR–dihydroartemisinin complex, the RMSD results showed (Figure 6C) that the protein backbone rose rapidly at the initial stage of the simulation, then gradually stabilized at about 20 ns, and the RMSD value remained stable at about 1.25 nm in the subsequent 80 ns, indicating that the overall conformation of the complex reached a dynamic equilibrium during the simulation without large conformational fluctuations. The RMSF analysis showed (Figure 6A) that the fluctuation level of amino acid residues was generally low and stable, without obvious high fluctuation regions, indicating that the protein still maintained good conformational flexibility after binding to the small molecules, and no abnormal violent movement occurred in the key binding region, suggesting that the binding mode of small molecules and proteins was stable. The radius of gyration Rg decreased slightly at the initial stage of the simulation, then stabilized at about 3.6 nm after 20 ns, indicating that the overall protein peptide chain maintained a compact and stable folding state without obvious unfolding or loosening (Figure 6E).

The kinetic performance of the EGFR–cinnamic acid complex also showed good stability: its RMSD curve entered the plateau period after 30 ns and stabilized in the range of 1.0–1.25 nm, and the overall fluctuation amplitude was slightly lower than the former, indicating that the conformation of the complex converged more rapidly (Figure 6D). The RMSF results also showed that the overall fluctuation level of residues was low, and there was no abnormal high flexibility in the key binding pocket, verifying the stable binding of small molecules and EGFR (Figure 6B). The radius of gyration Rg also tended to be stable during the simulation, remaining at about 3.6 nm, indicating that the compactness of the overall protein structure was not affected, and the peptide chain always maintained a stable folding state (Figure 6F).

Based on the results of the three indicators, the complexes formed by dihydroartemisinin and cinnamic acid with EGFR both showed good structural stability within 100 ns simulation time. The RMSD, RMSF, and Rg curves could converge rapidly and remain stable, supporting the predicted stability of the binding mode between small molecules and EGFR under simulated physiological conditions and providing a reliable dynamic structural basis for their interaction and potential regulatory functions.

### 3.9. WYS Inhibits Cell Proliferation

**Figure 7:**
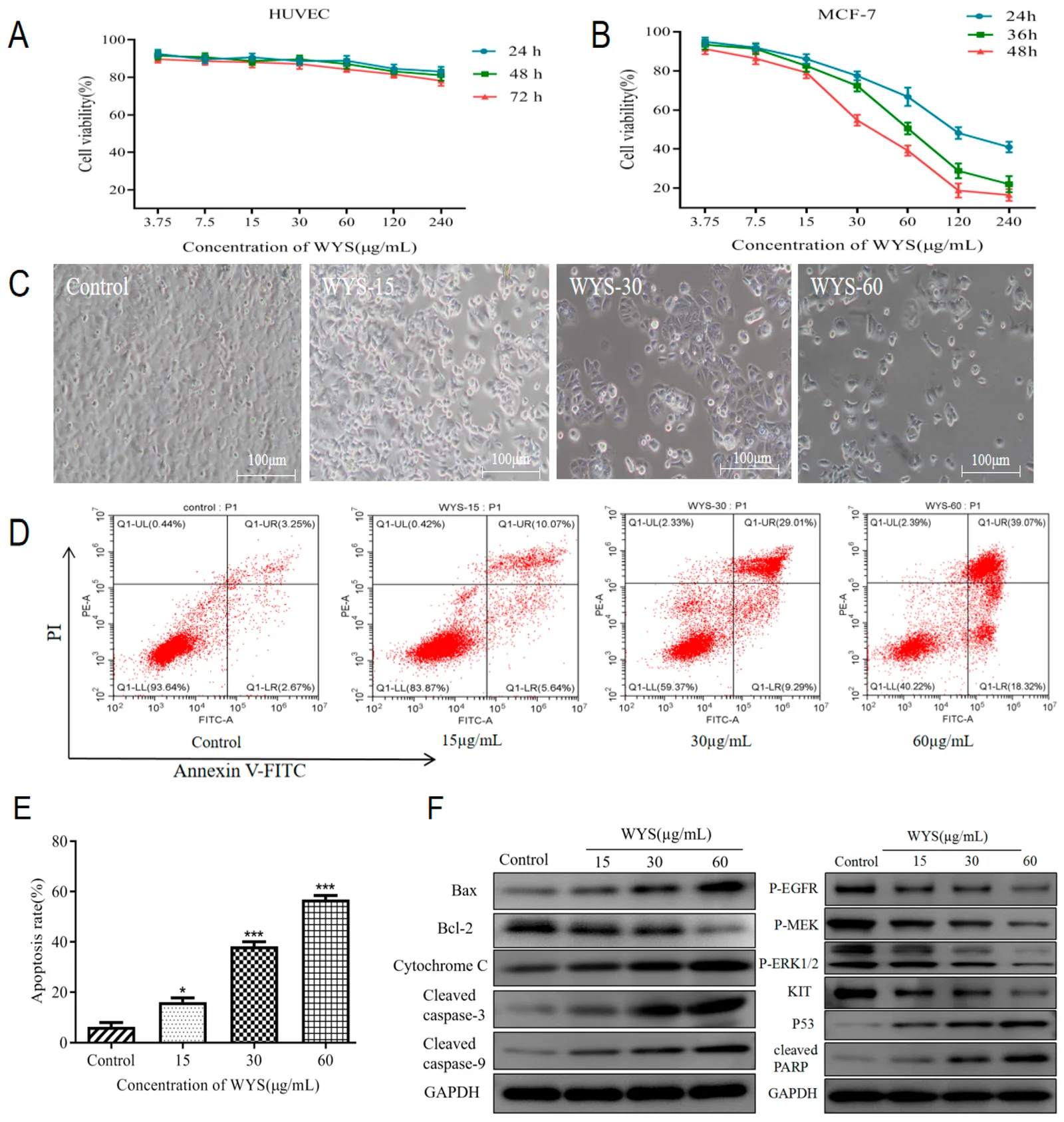
Effects of WYS on the proliferation and apoptosis of MCF-7 breast cancer cells. (**A**) Cell viability of HUVEC cells treated with different concentrations of WYS (3.75–240 μg/mL) for 24, 48, and 72 h, as determined by the MTT assay. (**B**) Cell viability of MCF-7 cells treated with different concentrations of WYS (3.75–240 μg/mL) for 24, 48, and 72 h, as determined by the MTT assay. (**C**) Morphological changes of MCF-7 cells after treatment with WYS (0, 15, 30, 60 μg/mL) for 48 h (scale bar = 100 μm). (**D**,**E**) Flow cytometric analysis of apoptosis in MCF-7 cells treated with WYS (0, 15, 30, and 60 μg/mL) for 48 h using Annexin V-FITC/PI double staining. The apoptosis rate was quantified in (**E**). Data are presented as the mean ± SD (*n* = 3). * *p* < 0.05, *** *p* < 0.001 vs. the control group. (**F**) Western blot analysis of apoptosis-related proteins (Bcl-2, Bax, cytochrome C, cleaved caspase-3, cleaved caspase-9, and cleaved PARP) and EGFR/MEK/ERK signaling pathway proteins (P-EGFR, P-MEK, P-ERK1/2, and P53) in MCF-7 cells treated with WYS (0, 15, 30, 60 μg/mL) for 48 h. GAPDH was used as the internal reference.

After treatment of HUVEC cells with different concentrations (3.75–240 μg/mL) of WYS for 24 h, 48 h, and 72 h, there was no significant change in cell survival rate (Figure 7A). At the same administration concentration, the inhibitory effect on breast cancer MCF-7 cells was significantly enhanced with increasing treatment duration (24 h < 36 h < 48 h), and the longer the action time, the more significant the anticancer effect. Under the 48 h long-term intervention (Figure 7B), medium and high concentrations of WYS could strongly inhibit the survival of breast cancer cells, with an IC_50_ of 45.8 μg/mL. WYS could significantly inhibit the proliferation of breast cancer MCF-7 cells in a dose- and time-dependent manner, showing selective cytotoxicity toward tumor cells, with no overt cytotoxic effect on HUVEC cells under the tested conditions.

### 3.10. WYS Induces Cell Apoptosis

Under an inverted microscope, the cells in the control group were morphologically intact, well-adherent, uniformly polygonal or spindle-shaped, tightly connected, and active. As the WYS concentration increased (15, 30, 60 μg/mL), cell adhesion gradually decreased, and the number of apoptotic cells exhibiting shrinkage, floating, and abnormal morphology increased in a dose-dependent manner (Figure 7C), suggesting that WYS could significantly damage cell morphology and induce apoptosis.

The results of Annexin V-FITC/PI double-staining flow detection showed that the proportion of viable MCF-7 cells in the blank control group was 93.64%, and the total spontaneous apoptosis rate was only 3.69%. After gradient intervention with 15, 30, and 60 μg/mL WYS, the proportion of viable cells decreased to 83.87%, 59.37%, and 40.22%. Meanwhile, the proportion of early apoptotic cells increased significantly from 3.25% to 10.07%, 29.01%, and 30.39%, the total apoptosis rate rose to 10.49%, 31.34%, and 48.71% synchronously, and the proportion of late apoptotic cells gradually increased with increasing WYS concentration (Figure 7D,E). The results confirmed that WYS could significantly induce programmed apoptosis of MCF-7 breast cancer cells in a concentration-dependent manner and inhibit the survival of tumor cells by activating the cell apoptosis pathway.

WB verification of apoptosis-related proteins in the cell apoptosis pathway showed (Figure 7F) that the anti-apoptotic protein Bcl-2 was highly expressed in the control group, while the pro-apoptotic protein Bax, cytochrome C, and cleaved caspase-3/9 (apoptosis executive proteins) were expressed at low levels. With increasing WYS concentration, the expression of Bcl-2 continued to decrease, the expression of Bax increased significantly, the release of mitochondrial cytochrome C increased, and the protein levels of cleaved caspase-3/9 increased in a concentration-dependent manner, confirming that WYS induced cell apoptosis by activating the mitochondria-mediated intrinsic apoptotic pathway. At the same time, after treatment with WYS, the phosphorylation levels of P-EGFR, P-MEK, and P-ERK1/2 decreased significantly in turn, and the protein levels of P53 and cleaved PARP increased synchronously, suggesting that WYS inhibited the activation of EGFR and its downstream MEK/ERK signaling axis, thereby regulating the P53-mediated apoptosis-related pathway and finally triggering cell apoptosis.

### 3.11. WYS Arrests Cell Cycle in G2/M Phase

**Figure 8:**
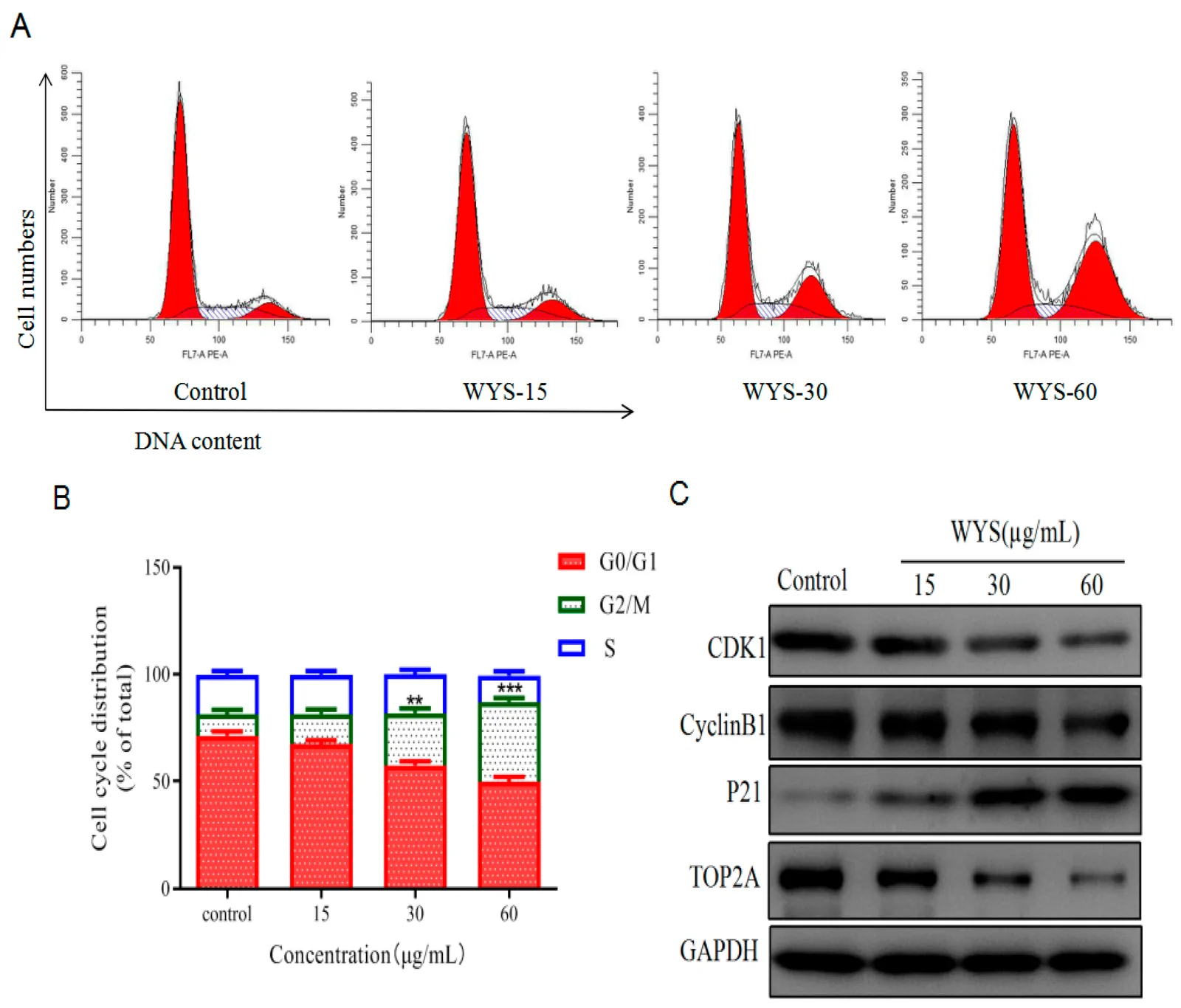
WYS induces G2/M phase arrest, as determined by flow cytometry and Western blot. (**A**) Representative histograms of cell cycle distribution analyzed by flow cytometry (*n* = 3 biological replicates per group). (**B**) Quantitative analysis of cell cycle distribution. Data are presented as mean ± SD, and a one-way ANOVA was used for comparison. Significance levels: ** *p* < 0.01, *** *p* < 0.001 vs. the control group (0 μg/mL WYS). (**C**) Western blot analysis of cell cycle-related proteins (CDK1, P21, Cyclin B1) (*n* = 3 biological replicates per group).

The results of flow cytometry DNA cycle analysis (Figure 8A,B) showed that the proportion of cells in the G0/G1 phase was the highest in the blank control group, and the proportion of cells in the G2/M phase was at a low basic level. With the gradual increase in WYS concentration (15, 30, 60 μg/mL), the proportion of cells in the G0/G1 phase continued to decrease significantly, the proportion of cells in the G2/M phase gradually increased with increasing WYS concentration, and the proportion of cells in the S phase showed no obvious regular fluctuation. Specifically, G2/M phase arrest in the 30 μg/mL treatment group was statistically significant (*p* < 0.01), and the accumulation of cells in G2/M phase in the 60 μg/mL high-concentration group reached an extremely significant level (*p* < 0.001). The shape of the flow cytometry histogram also intuitively showed that the cell population with G2/M phase DNA content was continuously enriched with increasing drug concentration, clearly indicating that WYS specifically blocked the cell cycle at the G2/M checkpoint, preventing cells from completing mitosis and rendering them unable to progress normally through the proliferation and division cycles. The Western blot protein detection results (Figure 8C) further clarified the molecular mechanism of cycle arrest: compared with the control group, the expression levels of CyclinB1,CDK1, and TOP2A, the key positive driving proteins of the G2/M cycle, continued to decrease with increasing drug concentration after WYS treatment, while the expression of P21, the negative regulatory checkpoint protein of the cycle, increased significantly in a concentration-dependent manner. The CyclinB1-CDK1 complex is the core molecular switch that drives cells across the G2/M checkpoint into the division phase. WYS blocks the transition from the G2/M phase to the division phase by inhibiting the expression of CyclinB1/CDK1 protein and activating the P21 cycle inhibition pathway, and, finally, stably induces cell G2/M cycle arrest, inhibiting the infinite proliferation and division of tumor cells.

### 3.12. WYS Alters Mitochondrial Morphology and Impairs Membrane Potential Homeostasis in Cancer Cells

**Figure 9:**
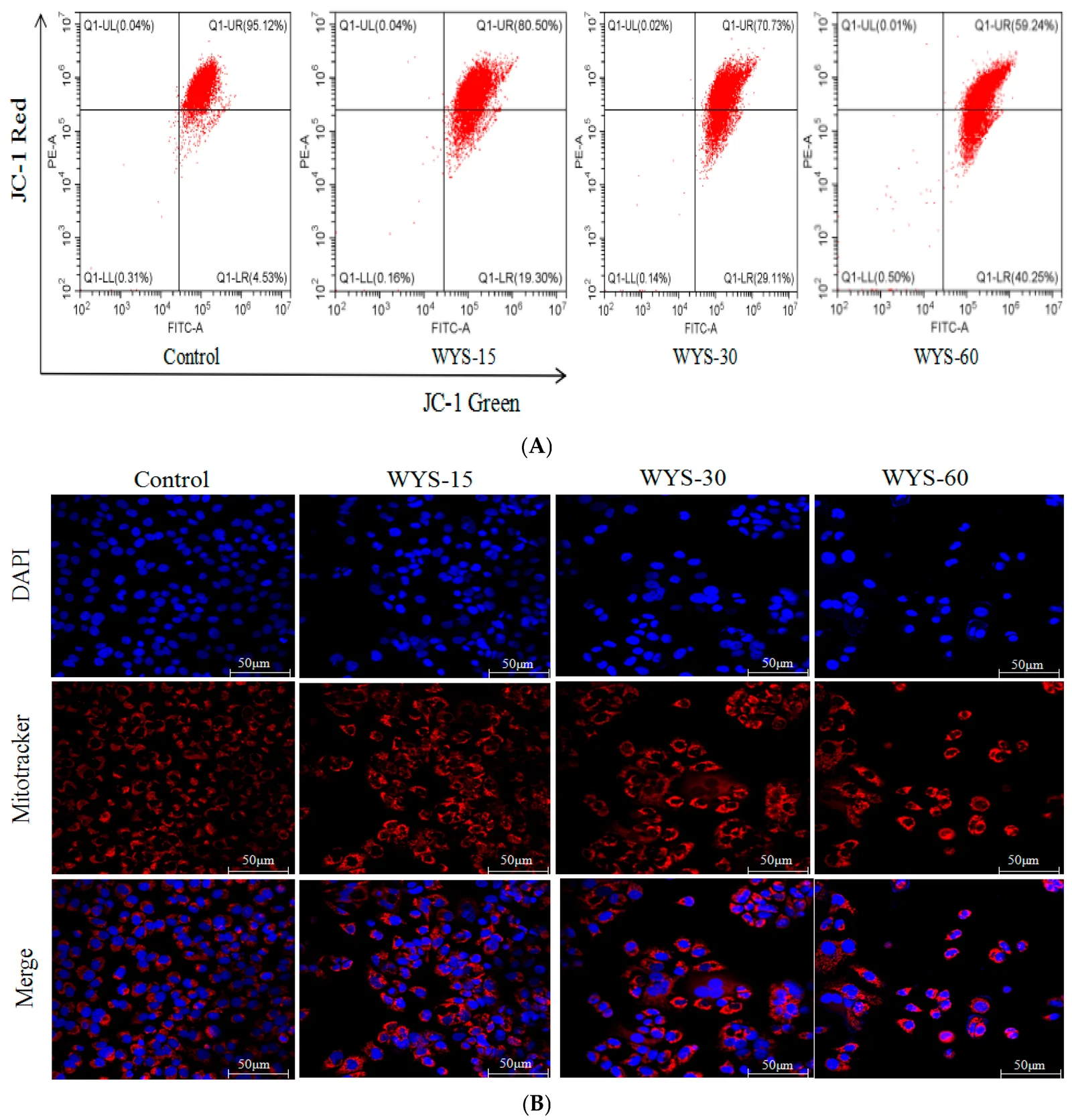
Effects of WYS on mitochondrial morphology and function in cells. (**A**) Scatter plots of mitochondrial membrane potential detected by JC-1 staining combined with flow cytometry. (**B**) Fluorescence microscopy images of cells co-stained with MitoTracker and DAPI (scale bar: 50 μm).

To further clarify the effect of WYS on mitochondrial function, we performed JC-1 flow detection and mitochondrial fluorescence staining. JC-1 flow results (Figure 9A) showed that the mitochondria of control cells maintained a high level of membrane potential, and the proportion of high red fluorescence quadrant was as high as 95.12%; with the increase in the WYS concentration, the proportion of red fluorescence cells continued to decrease, and the proportion of green fluorescence apoptotic cells increased significantly. The proportion of low membrane potential cells in the 60 μg/mL group reached 40.29%, confirming that WYS induced mitochondrial membrane potential depolarization and transmembrane potential gradient collapse in a dose-dependent manner. The MitoTracker immunofluorescence results (Figure 9B) showed that the mitochondria in the control group were uniformly and regularly distributed in a network-like pattern with a complete structure. Following WYS treatment, the mitochondria gradually exhibited disordered fluorescence, structural disruption, fragmentation, and aggregation, while at high WYS concentrations, the mitochondrial network structure completely collapsed and became diffusely and disorderly distributed. Combined with the DAPI nuclear staining results, it was confirmed that WYS significantly altered mitochondrial distribution and morphology and disrupted mitochondrial network homeostasis. Combined with the previous apoptosis results, it was revealed that WYS exerted anti-tumor cell activity through dual pathways of blocking cell cycle and inducing mitochondrial apoptosis.

### 3.13. WYS Has a Significant Inhibitory Effect on Tumors in Vivo

**Figure 10:**
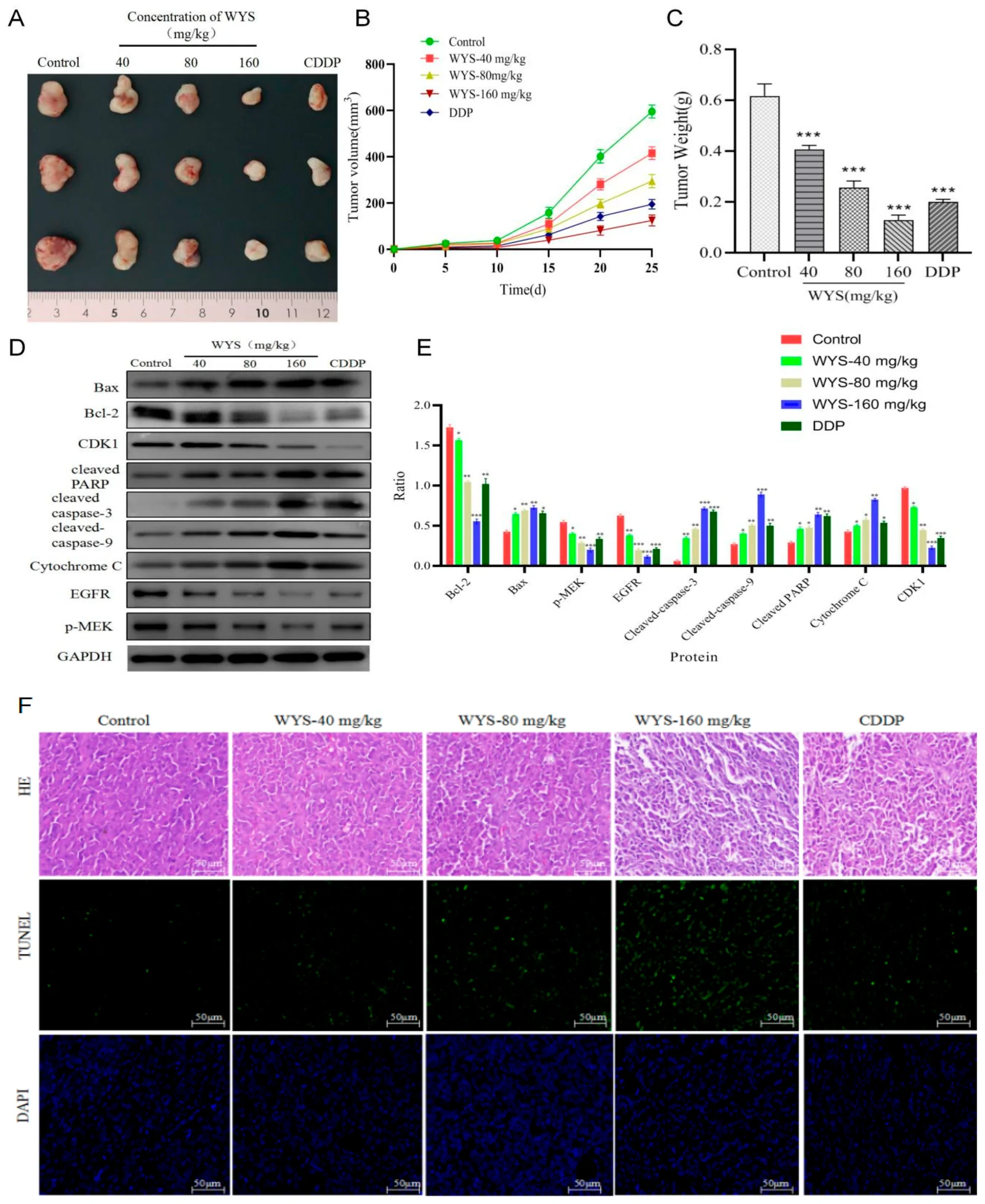
Inhibitory effect and mechanism of WYS on xenograft tumor growth in nude mice. (**A**) Representative images showing reduced tumor volume in WYS-treated mice. (**B**) Tumor volume growth curves during the treatment period. (**C**) Tumor weight of each group at the end of administration. (**D**,**E**) Western blot analysis and quantitative analysis of apoptosis- and proliferation-related proteins in tumor tissues (GAPDH was used as an internal reference). (**F**) H&E staining and TUNEL apoptosis staining of tumor tissues (scale bar: 50 μm). Cisplatin (CDDP) was used as the positive control. Data are presented as mean ± SD. * *p* < 0.05, ** *p* < 0.01, *** *p* < 0.001 vs. the control group.

Intuitive observation of isolated tumors showed that compared with the blank control group, the tumor volume of each WYS treatment group was significantly reduced, and the tumor size gradually decreased with the increase of administration dose (40, 80, 160 mg/kg). The tumor size of the high-dose 160 mg/kg group was very close to that of the cisplatin positive control group (Figure 10A); the tumor growth curve showed that with the extension of the administration cycle, the tumor in the control group continued to proliferate rapidly, while the tumor growth rate of each WYS dose group was significantly slowed down. The higher the dose, the stronger the anti-proliferation effect. At the 25 d observation end point, the tumor volume growth of the high-dose WYS group was significantly suppressed (Figure 10B); tumor weight statistics further quantitatively confirmed that the tumor weight of each WYS treatment group was significantly lower than that of the control group (*p* < 0.001), also showing a clear dose-dependent effect. The anti-tumor level of 160 mg/kg WYS was not significantly different from that of the cisplatin group (Figure 10C). Body weight monitoring showed no significant body weight loss in all WYS treatment groups during the experiment, indicating good in vivo tolerability of WYS at the tested doses (Supplementary Figure S2). The HE staining and TUNEL fluorescence apoptosis detection results further confirmed the in vivo anti-tumor effect of WYS at the histopathological level. The HE staining results showed that the tumor cells in the control group were densely and regularly arranged, with intact and uniform nuclear morphology, few atypical structures, dense tissue structure, and no obvious necrosis and apoptosis characteristics. With increasing WYS dose, tumor cell arrangement became increasingly disordered, cellular structure damage increased, nuclear pyknosis and fragmentation increased, and areas of interstitial necrosis increased. The pathological damage phenotype of the 160 mg/kg high-dose WYS group was highly consistent with that of cisplatin (CDDP)-positive drug. The TUNEL immunofluorescence staining results showed that there was only a very small amount of green apoptosis fluorescence signal in the control group, and the level of cell apoptosis was very low. Following the WYS intervention, the green apoptosis positive signal increased significantly in a dose-dependent manner, and the fluorescence intensity continued to rise. The density and fluorescence intensity of the apoptotic cells in the high-dose group were similar to those in the cisplatin positive control group. Combined with DAPI nuclear staining, the nuclear distribution characteristics of apoptotic damaged cells could be clearly observed (Figure 10F). The results of the Western blot detection of tumor tissues (Figure 10D,E) were completely consistent with those of the in vitro cell experiments: after WYS intervention, the expression of anti-apoptotic protein Bcl-2 in tumor tissues decreased, the levels of pro-apoptotic proteins Bax, cytochrome C, cleaved caspase-3/9, and cleaved PARP increased significantly, the expression of cycle key protein CDK1 was inhibited, and the phosphorylation levels of EGFR and its downstream p-MEK continued to decrease. It was clear that WYS also exerted efficient anti-tumor effects in vivo by activating the mitochondrial intrinsic apoptotic pathway, blocking the EGFR/MEK proliferation signaling pathway, blocking tumor cell cycle and inducing apoptosis.

## 4. Discussion

Medicinal fungi are generally recognized to have multi-target regulatory characteristics and relatively favorable biosafety profiles compared with single-target chemotherapeutic agents, making them excellent resources for developing novel anti-breast cancer drugs [31]. *I. levis* is an important medicinal fungus traditionally used for adjuvant treatment of tumors and diabetes in southern Xinjiang, China. Previous research on *I. levis* was limited to taxonomic identification and preliminary reports on polysaccharide components, lacking systematic analysis of its small-molecule metabolome and pharmacological mechanism. In this study, 152 metabolites were identified from a polysaccharide-free mycelium aqueous extract using UPLC-Q-TOF-MS, including phenylpropanoids, terpenoids, steroids, nucleosides, and other active substances, with dihydroartemisinin, cortisol, cinnamic acid, and guanosine as candidate core metabolites. Dihydroartemisinin, a classic sesquiterpene lactone, exerts potent anti-tumor activity in triple-negative breast cancer by inducing ferroptosis, inhibiting the EGFR/MEK/ERK pathway, and reversing chemotherapy resistance [32,33]. Cortisol, an endogenous glucocorticoid, plays a key regulatory role in hormone receptor-positive breast cancer by regulating GR-ER interactions, inhibiting cycle kinases and enhancing sensitivity to endocrine therapy [34,35]. Cinnamic acid and its derivatives are natural multi-drug resistance reversers, inhibiting P-glycoprotein, blocking the MAPK pathway, and suppressing tumor migration [36]. Guanosine exerts dual anti-tumor and chemotherapy-sensitizing effects in breast cancer by inducing tumor cell cycle arrest, remodeling the immune microenvironment, and reducing chemotherapy-induced inflammatory injury [37]. The coexistence of these diverse active components in WYS suggests that the anti-tumor effect of WYS may be the result of the synergistic action of multiple small molecules. This characteristic not only conforms to the holistic regulatory advantage of natural products but also avoids the limitations of single-target chemical drugs such as easy drug resistance, narrow spectrum of action, and strong toxic side effects to a certain extent [38]. Meanwhile, this study found that WYS had no significant toxicity to normal HUVEC cells at effective anti-tumor concentrations, demonstrating good selective killing ability against tumor cells. Comparative analysis within the *Inocutis* genus shows that *I. tamaricis* is reported to be rich in flavonoids, with mainly antioxidant activity, while *I. levis* in this study contains more terpenoids, steroids, and nucleosides, with prominent anti-proliferative and cell cycle regulatory activities, reflecting species-specific metabolic characteristics. This study is the first to confirm that *I. levis* mycelium is rich in highly active anti-tumor small molecules, challenging the traditional view that polysaccharides are the active basis of this species and thereby significantly enhancing its medicinal development potential and scientific value.

In terms of target screening, five core characteristic genes, CDK1, TOP2A, EGFR, FGF2, and KIT were finally obtained through multi-database cross-integration, TCGA differential gene analysis, PPI network construction, and combined screening using three machine learning algorithms. These targets are key drivers of breast cancer occurrence, development, proliferation, invasion, and drug resistance. EGFR amplification or mutation is an important driver of triple-negative breast cancer and HER2-negative breast cancer progression and is directly related to endocrine and targeted drug resistance [39]. CDK1, a key cell cycle kinase, is closely associated with poor prognosis and chemotherapy resistance in breast cancer [40]. GO and KEGG enrichment analyses showed that the targets were significantly enriched in core breast cancer pathways such as cell cycle regulation, mitochondrial apoptosis, the EGFR tyrosine kinase pathway, MAPK signaling, and PI3K-Akt signaling, indicating that WYS does not act randomly on cells but precisely targets the key regulatory networks of breast cancer proliferation–cell cycle–apoptosis–invasion, which are highly consistent with the core pathways of clinical targeted therapy [41]. This fully demonstrates the high biological significance and reliability of the target screening results in this study. Molecular docking and 100 ns molecular dynamics simulations further indicated that the core metabolites in WYS can form stable binding with key targets. This elevates the predictive relationships of network pharmacology to direct molecular interactions, significantly enhancing the reliability and rigor of the mechanism conclusions in this study.

An uncontrolled cell cycle is the core mechanism of malignant proliferation in breast cancer [42]. This study found that WYS induces G2/M phase arrest in MCF-7 cells in a concentration-dependent manner, significantly downregulating CyclinB1 and CDK1 expression and upregulating P21 levels. The CDK1/CyclinB1 complex is an essential molecular switch for cells to pass the G2/M checkpoint, and its inhibition directly blocks mitosis, while P21, a broad-spectrum cycle inhibitor, induces cycle arrest by binding to the CDK1 complex [43]. Studies have confirmed that various natural products such as curcumin, resveratrol, and *Phellinus igniarius* flavonoids exert anti-breast cancer effects by targeting CDK1 and inducing G2/M arrest [44]. This study extends this key mechanism to *I. levis* and confirms that the cycle arrest effect is highly consistent in vitro and in vivo.

Apoptosis evasion is an important cause of breast cancer recurrence, metastasis, and chemotherapy resistance [45]. This study confirmed that WYS induces apoptosis in MCF-7 cells via the mitochondria-dependent endogenous apoptotic pathway from multiple aspects: cell morphology, flow cytometry, mitochondrial membrane potential, and apoptotic protein expression. JC-1 assay showed that WYS significantly reduced mitochondrial membrane potential, and MitoTracker fluorescence revealed fragmented mitochondrial structure and disrupted network homeostasis. Meanwhile, the expression of the pro-apoptotic protein Bax was upregulated, the anti-apoptotic protein Bcl-2 was downregulated, cytochrome C was released in large quantities, and cleaved caspase-9, cleaved caspase-3, and cleaved PARP were activated, initiating the apoptotic cascade. Studies have reported that both EGFR inhibitors and CDK1 inhibitors trigger apoptosis by disrupting mitochondrial homeostasis [46], which is consistent with the results of this study. Moreover, WYS selectively damages tumor cell mitochondria with minimal impact on normal cells, an advantage superior to most traditional chemotherapeutic drugs.

**Figure 11:**
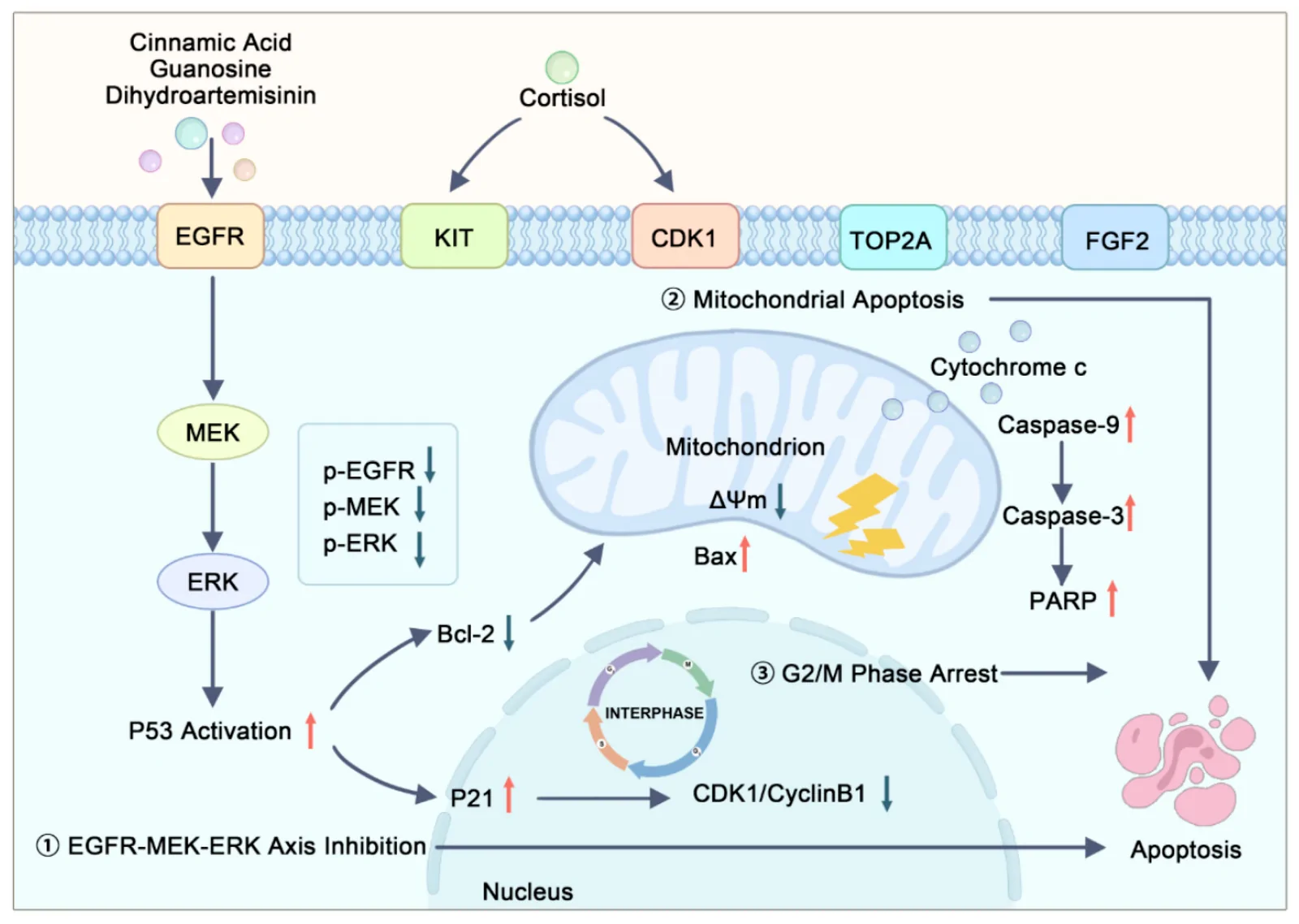
Proposed molecular mechanism of WYS extracted from *I. levis* in suppressing breast cancer.

Further mechanistic studies revealed that inhibition of the EGFR/MEK/ERK signaling axis may be an important upstream regulatory link for WYS to exert anti-breast cancer effects. In vitro and in vivo experiments consistently showed that WYS significantly reduced the levels of p-EGFR, p-MEK, and p-ERK1/2 and upregulated the expression of the tumor suppressor gene P53. Abnormal activation of EGFR is an important driver of breast cancer, especially triple-negative breast cancer, and excessive activation of its downstream MEK/ERK pathway promotes proliferation, inhibits apoptosis, enhances invasion and induces endocrine resistance. Clinical EGFR inhibitors are prone to drug resistance due to secondary mutations [47,48]. In contrast, WYS targets multiple key nodes such as EGFR, CDK1, and TOP2A simultaneously with multiple components, blocking proliferation signal input upstream, arresting the cell cycle midstream, and activating apoptosis downstream, forming a multi-node synergistic regulatory pattern. Meanwhile, P53 plays a key bridging role in this pathway. Inhibition of EGFR/ERK relieves inhibition of P53, which in turn transcriptionally activates downstream molecules such as P21 and Bax, simultaneously achieving cycle arrest and apoptosis activation (Figure 11). This dual regulatory pattern of inhibiting oncogenic signaling and activating tumor suppressor pathways is difficult for single-target drugs to achieve [49,50].

In vivo nude mouse xenograft tumor experiments provided key evidence for the anti-breast cancer efficacy of WYS. The results showed that WYS inhibited tumor growth in a dose-dependent manner, with anti-tumor efficacy comparable to cisplatin at high doses. HE staining revealed significant nuclear pyknosis and cell death in tumor cells, TUNEL fluorescence staining showed a significant increase in apoptotic cells, and tumor tissue protein detection results were completely consistent with in vitro experiments. This confirmed that WYS can stably and reproducibly induce cell cycle arrest, activate apoptosis and inhibit pathway signaling in vivo, providing preliminary experimental support for future investigation of *I. levis*-derived bioactive fractions as potential natural anti-tumor agents.

Despite the comprehensive nature of these findings, several limitations persist within this study. Firstly, Only MCF-7 cells were used as a model, lacking coverage of triple-negative breast cancer, HER2-positive and other subtypes, and the universality of its action needs to be expanded. Secondly, The four candidate core metabolites have not been isolated and purified for individual activity verification and target validation. The synergistic ratio and key effector molecules of each component require further detailed analysis. Thirdly, key data on pharmacokinetics, long-term toxicity and tissue distribution for drug development are lacking. This screening criterion may exclude some low-abundance but potentially bioactive metabolites. Future research can focus on verifying the targets of monomers such as dihydroartemisinin, cortisol and cinnamic acid via knockout and overexpression models, expanding research to drug-resistant strains and PDX models to enhance clinical relevance, combining activity-guided fractionation and sensitive targeted metabolomics to explore minor bioactive components in WYS, to obtain a more comprehensive understanding of its material basis. conducting pharmacokinetic, toxicity and formulation process research, and exploring the synergistic efficacy and drug resistance reversal of WYS combined with chemotherapy, targeted therapy and immunotherapy, further improving its anti-breast cancer regulatory network.

## 5. Conclusions

The active fraction WYS from *I. levis* mycelium is an efficient, low-toxicity and multi-target natural anti-breast cancer active component. The current findings suggest that small molecules such as dihydroartemisinin, cortisol, guanosine and cinnamic acid may interact with key proteins including EGFR, CDK1, TOP2A, FGF2 and KIT as predicted by computational analysis, inhibit the EGFR/MEK/ERK oncogenic signaling pathway, downregulate CyclinB1/CDK1 and upregulate P21 to induce G2/M cell cycle arrest, and simultaneously disrupt mitochondrial membrane homeostasis to initiate the endogenous apoptotic pathway, ultimately significantly inhibiting breast cancer growth in vitro and in vivo. The findings provide promising preclinical evidence supporting further exploration of *I. levis* as food-derived bioactive resources. Further mechanistic, toxicological and in-depth efficacy studies are still warranted to evaluate their translational potential as functional food ingredients for dietary adjuvant intervention against breast cancer.

## Figures and Tables

**Table 1:** Molecular docking binding energy.

Gene	Metabolite	Pubchem CID	Affinity (kcal/mol)
CDK1	Cortisol	5754	−8.104
EGFR	Cinnamic acid	444539	−5.474
EGFR	Dihydroartemisinin	3000518	−7.214
EGFR	Guanosine	135398635	−7.209
KIT	Cortisol	5754	−7.503
TOP2A	Dihydroartemisinin	3000518	−7.343

## Data Availability

The original contributions presented in this study are included in the article/Supplementary Material. Further inquiries can be directed to the corresponding authors.

## Supplementary Materials

The following supporting information can be downloaded at: https://www.mdpi.com/article/10.3390/foods15183199/s1, Supplementary Table S1: 72 key metabolites screened under positive ion mode. Supplementary Table S2: 89 key metabolites screened under negative ion mode. Supplementary Figure S1: GSEA Analysis of Characteristic Genes. Supplementary Figure S2: Body weight changes of BALB/c nude mice during treatment.

## Abbreviations

MTT	3-(4,5-dimethylthiazol-2-yl)-2,5-diphenyltetrazolium bromide
PPI	Protein–protein interaction
GO	Gene ontology
KEGG	Kyoto encyclopedia of genes and genomes
ROC	Receiver operating characteristic
GSEA	Gene set enrichment analysis
RMSD	Root mean square deviation
RMSF	Root mean square fluctuation
Rg	Radius of gyration
HUVEC	Human umbilical vein endothelial cells
ΔΨm	Mitochondrial membrane potential
JC-1	5,5′,6,6′-Tetrachloro-1,1′,3,3′-tetraethyl- imidacarbocyanine iodide
TUNEL	Terminal deoxynucleotidyl transferase-mediated dUTP nick end labeling
HE	Hematoxylin-eosin
CDDP	Cisplatin
SPF	Specific pathogen-free
LASSO	Least absolute shrinkage and selection operator
SVM-RFE	Support vector machine-recursive feature elimination
MSI	Metabolomics Standards Initiative
PDX	Patient-derived xenograft
