# Supplementary material for: Inocutis levis Mycelial Extract WYS Exerts Anti-Proliferative and Pro-Apoptotic Effects on MCF-7 Cells via EGFR/MEK/ERK Modulation and G2/M Arrest
Source: Foods. 2026 Sep 10;15(18):3199. doi: 10.3390/foods15183199 (PMC13605065; doi:10.3390/foods15183199)
Supplement: Supplementary file 1 [file foods-15-03199-s001.zip › Supplementary Figure S1.pdf]

**Supplementary Figure S1: GSEA Analysis of Characteristic Genes**

As described in the Methods section, GSEA was performed on five characteristic genes, respectively.The CDK1 gene was enriched in 100 pathways, the TOP2A gene in 103 pathways, the EGFR gene in 102 pathways, the FGF2 gene in 105 pathways, and the KIT gene in 107 pathways.

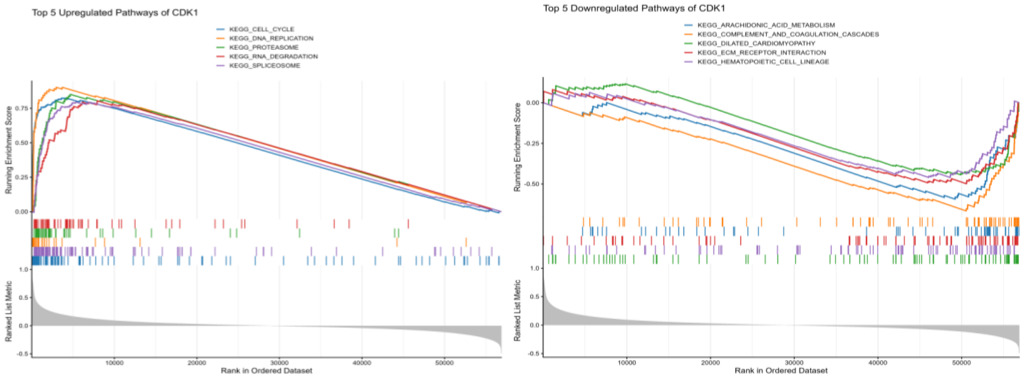


Figure S1-A GSEA analysis plot of the CDK1 gene

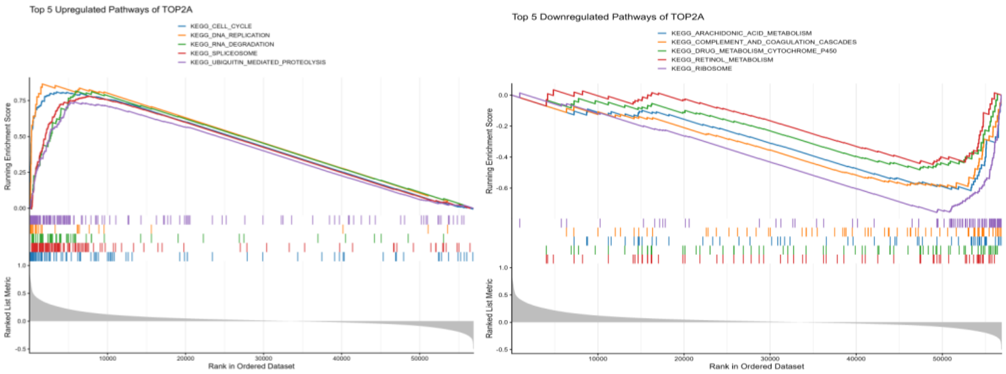


Figure S1-B GSEA analysis plot of the TOP2A gene

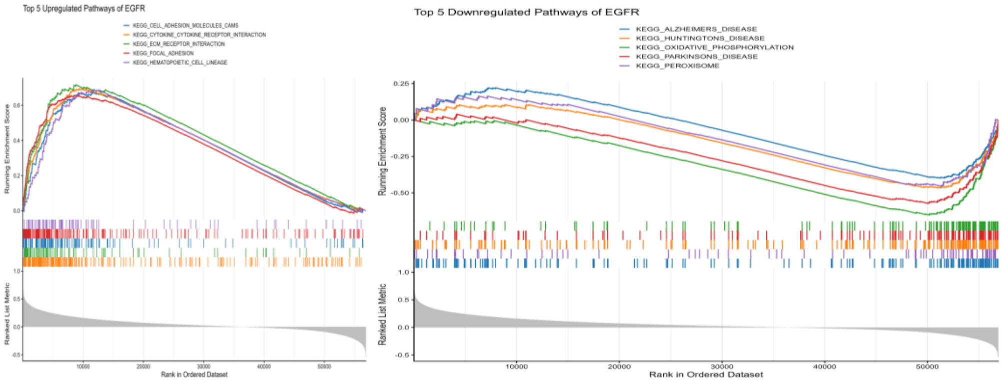


Figure S1-C GSEA analysis plot of the EGFR gene

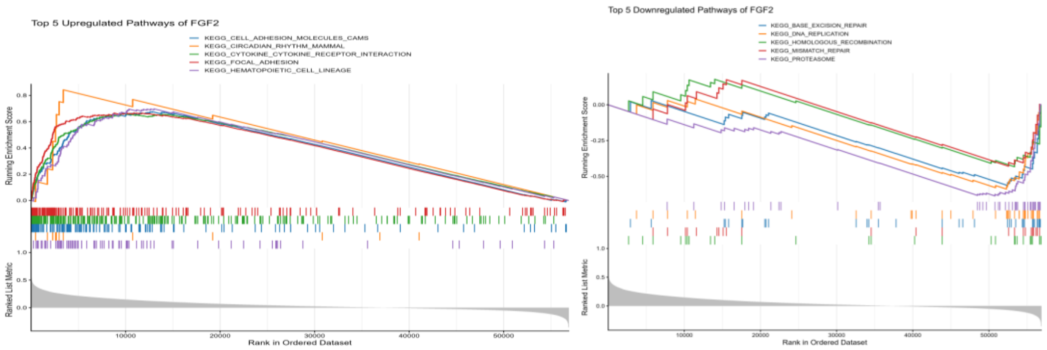


Figure S1-D GSEA analysis plot of the FGF2 gene

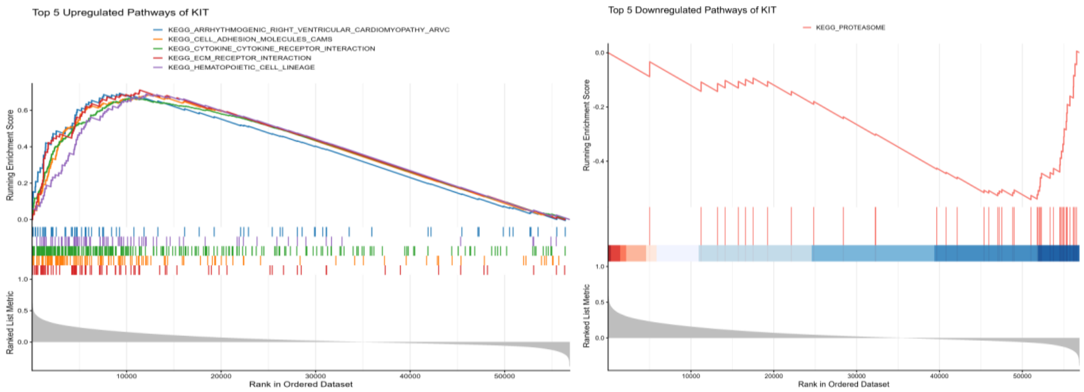


Figure S1-E GSEA analysis plot of the KIT gene

Note: The left panels show enrichment analysis plots of the top 5 upregulated pathways; the right panels show enrichment analysis plots of the top 5 downregulated pathways.The vertical axis in the upper half represents the NES score; values greater than 0 indicate upregulated pathways, and values less than 0 indicate downregulated pathways.The corresponding vertical lines in the middle represent genes; the denser the genes, the more enriched the pathway at this position. Lines and vertical lines of different colors correspond to different pathways and the pathway names in the legend.The lower half shows the calculated rank values, sorted in descending order.
