# Supplementary material for: Inocutis levis Mycelial Extract WYS Exerts Anti-Proliferative and Pro-Apoptotic Effects on MCF-7 Cells via EGFR/MEK/ERK Modulation and G2/M Arrest
Source: Foods. 2026 Sep 10;15(18):3199. doi: 10.3390/foods15183199 (PMC13605065; doi:10.3390/foods15183199)
Supplement: Supplementary file 1 [file foods-15-03199-s001.zip › Supplementary Figure S2.pdf]

Supplementary Figure S2: Body weight changes of BALB/c nude mice during treatment

Body weight monitoring showed no significant body weight loss in all WYS treatment groups throughout the 25-day experimental period (Supplementary 4). All WYS-treated groups maintained a steady growth trend consistent with the control group, indicating good in vivo tolerability of WYS at the tested doses. The CDDP positive control group showed a slight transient weight decrease on day 3, followed by gradual recovery, and the final body weight was very close to that of the WYS 160 mg/kg group.

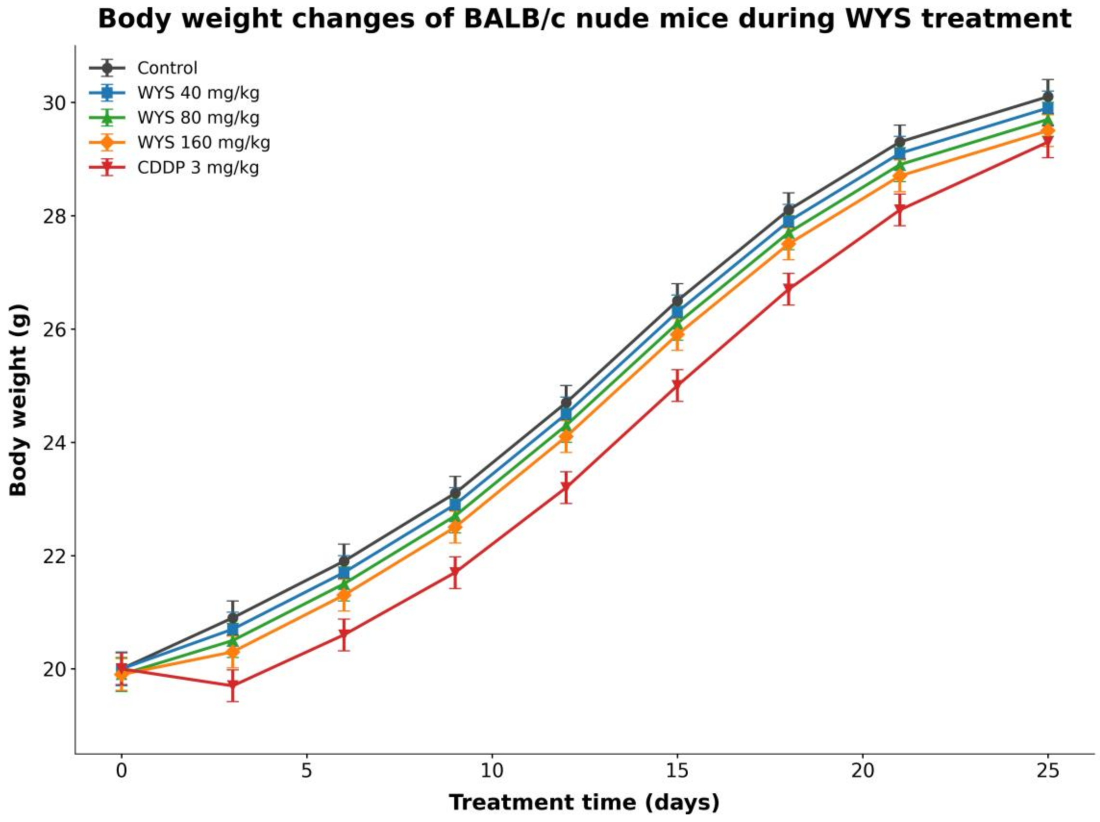

Supplementary Figure S2: Curve of body weight changes in mice. Data are expressed as mean ± SD (n = 6 mice per group). Mice were administered with normal saline (Control), 40, 80, 160 mg/kg WYS (intragastric) or 3 mg/kg cisplatin (CDDP, intraperitoneal) once every 3 days.
