# Supplementary material for: Inocutis levis Mycelial Extract WYS Exerts Anti-Proliferative and Pro-Apoptotic Effects on MCF-7 Cells via EGFR/MEK/ERK Modulation and G2/M Arrest
Source: Foods. 2026 Sep 10;15(18):3199. doi: 10.3390/foods15183199 (PMC13605065; doi:10.3390/foods15183199)
Supplement: Supplementary file 1 [file foods-15-03199-s001.zip › Supplementary Table S1.pdf]

Supplementary Table S1：72 key metabolites screened under positive ion mode

Number	Name	Calc. MW	RT [min]	Relative Peak Area
1	1,5-Naphthalenediamine	158.08	4.64	0. 105%
2	Parthenolide	248.14	10.09	0. 106%
3	Acetyl-β-methylcholine	159.13	1.14	0. 107%
4	L-Histidine	155.07	0.92	0. 112%
5	N6-Me-Adenosine	281.11	2.06	0. 115%
6	2-Aminooctanedioic acid	189.10	1.54	0. 127%
7	Guvacoline	141.08	2.52	0. 129%
8	Ginkgolic acid (C13:0)	320.23	11.63	0. 132%
9	Adenosine 3'5'-cyclic monophosphate	329.05	1.21	0. 138%
10	Bis(2,2-dihydroxyethyl) hydrogen phosphate	218.02	0.87	0. 152%
11	2'-Deoxyadenosine	251.10	1.35	0. 167%
12	Cyclo(leucylprolyl)	210.14	6.59	0. 168%
13	Acetyl-L-carnitine	203.12	1.21	0. 184%
14	L-Glutamic acid	147.05	0.96	0. 186%
15	Cortisol	362.21	10.70	0. 191%
16	D-(+)-Proline	115.06	4.56	0. 192%
17	2'-O-Methyladenosine	281.11	1.71	0. 194%
18	Bis(4-ethylbenzylidene)sorbitol	414.20	12.10	0. 196%
19	2-Pyridylacetic acid	137.05	5.18	0. 204%
20	α-Eleostearic acid	278.22	13.30	0. 212%
21	α-Aspartylphenylalanine	280.11	4.78	0. 212%
22	Prolylleucine	228.15	3.21	0. 235%
23	13(S)-HOTrE	294.22	11.13	0. 259%
24	DL-Lysine	146.11	0.80	0. 261%
25	Aspartame	294.12	2.60	0. 262%
26	D-(+)-Pipecolinic acid	129.08	0.92	0. 266%
27	2-Amino-1,3,4-octadecanetriol	317.29	10.69	0. 269%
28	8-{3-Oxo-2-[(2E)-2-penten-1-yl]-1-cyclopenten-1-yl}octanoic acid	292.20	10.26	0. 283%
29	Tolycaine	278.16	6.44	0. 294%
30	Hypoxanthine	136.04	1.22	0. 299%
31	Glycyl-L-leucine	188.12	1.68	0. 305%
32	Cytidine	243.08	1.13	0. 314%
33	Adenosine 5'-monophosphate	347.06	1.16	0. 337%
34	3,4-Dihydroxybenzaldehyde	138.03	5.16	0. 339%
35	Uracil	112.03	1.26	0. 388%

36	Argininosuccinic acid	290.12	1.16	0. 405%
37	Nicotinamide	122.05	1.22	0. 420%
38	Diethyl (2-nitro-1-phenylethyl)malonate	309.12	2.19	0. 545%
39	4-Methoxycinnamaldehyde	162.07	7.88	0. 550%
40	N-Acetyl-L-carnosine	268.12	1.20	0. 608%
41	7,8-Bis(hydroxymethyl)-1,4a-dimethyl-3,4,4a,5,6,7-hexahydro-2H-benzo[7]annulen-2-one	250.16	10.72	0. 654%
42	Nicotinic acid	123.03	1.05	0. 656%
43	Valylproline	214.13	1.69	0. 760%
44	4-Methoxycinnamic acid	178.06	6.92	0. 770%
45	1,2-di-O-methyl-4-[(2R)-2,4-dihydrobutyramido]-4,6-dideoxy-α-D-mannopyranoside	293.15	1.36	0. 855%
46	tetraethyl {2-[4-(tetradecyloxy)phenyl]ethane-1,1-diyl}bis(phosphonate)	590.35	6.64	0. 902%
47	DIETHYL (BOC-AMINO)MALONATE	275.14	1.37	0. 908%
48	Cytosine	111.04	1.00	0. 916%
49	Leucylproline	228.15	4.09	0. 922%
50	PEG n5	238.14	4.49	0. 979%
51	4-[5-Hydroxy-6-(2-hydroxy-3-methyl-4-methylenetetrahydro-2-furanyl)-1,3-dioxan-4-yl]-2,6-piperidinedione	327.13	2.19	0. 989%
52	2-Hydroxycinnamic acid	164.05	1.30	0. 995%
53	tert-Butyl N-[1-(aminocarbonyl)-3-methylbutyl]carbamate	230.16	4.27	1. 015%
54	Guanosine	283.09	1.40	1. 243%
55	Glycerophosphocholine	257.10	0.94	1. 450%
56	4-[(E)-(2-Amino-4-hydroxy-5-methylphenyl)diazenyl]-N-(2-pyridinyl)benzenesulfonamide	383.11	3.54	1. 670%
57	Betaine	117.08	0.91	2. 055%
58	4-Guanidinobutyric acid	145.08	1.18	2. 131%
59	Undecaethylene glycol	502.30	6.35	2. 230%
60	PEG n6	282.17	5.08	2. 324%
61	Guanine	151.05	1.40	2. 360%
62	DL-Arginine	174.11	0.91	2. 787%
63	L-Pyroglutamic acid	129.04	1.25	2. 849%

64	Decaethylene Glycol	458.27	6.17	3.217%
65	PEG n7	326.19	5.46	3.408%
66	D-(+)-Tryptophan	204.09	4.64	3.767%
67	PEG n8	370.22	5.74	3.956%
68	Nonaethylene Glycol	414.25	5.97	4.011%
69	Choline	103.10	0.89	4.280%
70	L-Isoleucine	131.09	1.41	9.934%
71	Phenylacetylene	102.05	2.26	11.995%
72	Cinnamic acid	148.05	2.26	13.170%

Note: Metabolites were putatively annotated based on exact mass matching (mass error < 5 ppm) and MS/MS spectral matching with public databases. Values are semi-quantitative estimates calculated by peak area normalization.
