# Supplementary material for: Inocutis levis Mycelial Extract WYS Exerts Anti-Proliferative and Pro-Apoptotic Effects on MCF-7 Cells via EGFR/MEK/ERK Modulation and G2/M Arrest
Source: Foods. 2026 Sep 10;15(18):3199. doi: 10.3390/foods15183199 (PMC13605065; doi:10.3390/foods15183199)
Supplement: Supplementary file 1 [file foods-15-03199-s001.zip › Supplementary Table S2.pdf]

Supplementary Table S2: 89 key metabolites screened under negative ion mode

Number	Name	Calc. MW	RT [min]	Relative Peak Area
1	1,2,3,4-Tetrahydro-3-isoquinolinecarboxylic acid	177.08	1.46	0. 102%
2	3-Hydroxy-3-(methoxycarbonyl)pentanedioic acid	206.04	1.92	0. 103%
3	Octyl gallate	282.15	8.87	0. 103%
4	(±)-Abscisic acid	264.14	10.64	0. 105%
5	3,12-Dihydroxy-13-methoxypodocarpa-8,11,13-trien-7-one	304.17	10.60	0. 107%
6	Esculetin	178.03	5.74	0. 108%
7	4-Hydroxybenzoic acid	138.03	4.80	0. 113%
8	5-Dehydroquinic acid	190.05	2.48	0. 118%
9	N2-Methylguanosine	297.11	1.92	0. 122%
10	16-Hydroxyhexadecanoic acid	272.24	16.15	0. 122%
11	2-Furoic acid	112.02	4.98	0. 128%
12	α-Aspartylphenylalanine	280.11	4.73	0. 133%
13	Homogentisic acid	168.04	3.08	0. 145%
14	2-(Acetylamino)hexanoic acid	173.10	6.31	0. 146%
15	1,2,4-Benzenetricarboxylic acid	210.02	6.31	0. 146%
16	4-Coumaric acid	164.05	4.93	0. 158%
17	D(+)-Phenyllactic acid	166.06	6.79	0. 165%
18	3-Isopropylmalic acid	176.07	1.70	0. 167%
19	1-(4-Fluorophenyl)-2-(phenylsulfonyl)ethanone	278.04	0.83	0. 174%
20	2'-Deoxyuridine	228.07	1.20	0. 174%
21	D-α-Hydroxyglutaric acid	148.04	0.99	0. 175%
22	N-Acetyl-L-phenylalanine	207.09	6.82	0. 190%
23	L-Tyrosine	181.07	1.24	0. 197%
24	trans-Cinnamic acid	148.05	2.22	0. 204%
25	4-[5-Hydroxy-6-(2-hydroxy-3-methyl-4-methylenetetrahydro-2-furanyl)-1,3-dioxan-4-yl]-2,6-piperidinedione	327.13	2.12	0. 207%
26	D-(-)-Ribose	150.05	0.83	0. 211%
27	N-Tigloylglycine	157.07	1.35	0. 220%
28	Adenosine	267.10	1.25	0. 222%
29	2-Hydroxyphenylacetic acid	152.05	5.34	0. 223%
30	6-Gingerol	294.18	11.41	0. 235%
31	Leucylproline	228.15	4.02	0. 237%
32	2-(8-Hydroxy-4a,8-dimethyldecahydro-2-naphthalenyl)acrylic acid	252.17	10.82	0. 247%

33	Isophthalic acid	166.03	6.33	0. 258%
34	2-Hydroxynicotinic acid	139.03	1.71	0. 265%
35	4-Indolecarbaldehyde	145.05	7.61	0. 269%
36	L-Histidine	155.07	0.74	0. 298%
37	1-Deoxy-1-{2,6,8-trioxo-7-[3-(phosphonooxy)propyl]-1,2,3,6,7,8-hexahydro-9H-purin-9-yl}-D-ribitol	440.09	0.84	0. 309%
38	6-Hydroxypicolinic acid	139.03	1.18	0. 313%
39	2-[(2S,4aR,8aS)-2-Hydroxy-4a-methyl-8-methylenedecahydro-2-naphthalenyl]acrylic acid	250.16	10.61	0. 326%
40	3-Methylglutaric acid	146.06	4.50	0. 337%
41	Phthalic acid	166.03	4.09	0. 342%
42	Suberic acid	174.09	6.65	0. 346%
43	2-(1,3-Dimethyl-2,6-dioxo-1,2,3,6-tetrahydro-purin-7-yl)-N,N-diethyl-acetamide	293.15	1.29	0. 348%
44	L-Gulono-1,4-lactone	178.05	1.22	0. 358%
45	cis-Aconitic acid	174.02	1.20	0. 373%
46	L-Glutamic acid	147.05	0.82	0. 387%
47	Xanthine	152.03	1.18	0. 406%
48	Glycyl-L-leucine	188.12	3.11	0. 424%
49	L-THREONIC ACID	136.04	0.83	0. 428%
50	etisulergine	376.19	10.20	0. 452%
51	2-Aminooctanedioic acid	189.10	5.36	0. 471%
52	Span(R) 20	346.24	8.58	0. 472%
53	Dihydroartemisinin	284.16	9.99	0. 480%
54	2-Methylbenzoic acid	136.05	5.81	0. 508%
55	Terephthalic acid	166.03	2.79	0. 568%
56	2,3-Dihydro-1-benzofuran-2-carboxylic acid	164.05	6.69	0. 575%
57	Citramalic acid	148.04	1.35	0. 581%
58	Dodecanedioic acid	230.15	10.35	0. 650%
59	3-Hydroxy-3-methylglutaric acid	162.05	1.44	0. 705%
60	3-tert-Butyladipic acid	202.12	8.80	0. 748%
61	Uridine monophosphate (UMP)	324.04	0.84	0. 794%
62	Porphobilinogen	226.10	2.66	0. 804%
63	2'-Deoxyguanosine 5'-monophosphate (dGMP)	347.06	1.08	0. 878%
64	Guanosine monophosphate (GMP)	363.06	1.12	0. 910%
65	3-Methyladipic acid	160.07	5.59	0. 914%
66	(+/-)9,10-dihydroxy-12Z-octadecenoic acid	314.25	12.24	0. 924%

67	2-Deoxy-2,3-dehydro-N-acetylneuraminic acid	291.10	0.88	1.000%
68	(+/-)12(13)-DiHOME	314.25	11.74	1.129%
69	(10E,12Z)-9-Hydroperoxy-10,12-octadecadienoic acid	312.23	10.92	1.150%
70	Uridine	244.07	1.21	1.167%
71	(1S,4R,4aS,8aS)-1,2-Bis(hydroxymethyl)-5,5,8a-trimethyl-1,4,4a,5,6,7,8,8a-octahydro-1,4-naphthalenediol	270.18	8.40	1.177%
72	Corchorifatty acid F	328.23	10.51	1.399%
73	Calcium pantothenate	219.11	2.86	1.409%
74	D-(+)-Tryptophan	204.09	4.60	1.446%
75	Guanosine	283.09	1.34	1.460%
76	decanediol dimethacrylate	310.21	11.50	1.492%
77	Caffeic acid	180.04	5.81	1.706%
78	Gentisic acid	154.03	3.95	1.707%
79	p-Acetoxystyrene	162.07	7.82	2.558%
80	Succinyladenosine	383.11	3.61	2.860%
81	Azelaic acid	188.10	7.57	3.172%
82	(−)-pinellic acid	330.24	10.50	3.449%
83	D-(-)-Mannitol	182.08	0.79	3.530%
84	L-Phenylalanine	165.08	2.22	3.697%
85	1,3,4,6-Tetra-O-acetyl-2-azido-2-deoxy-α-D-galactopyranose	373.11	1.20	4.109%
86	L-(-)-Arabitol	152.07	0.81	4.753%
87	3-Allyl-2-hydroxybenzoic acid	178.06	6.86	6.579%
88	2,5-Dihydroxybenzaldehyde	138.03	5.12	7.000%
89	(15Z)-9,12,13-Trihydroxy-15-octadecenoic acid	330.24	9.94	17.086%

Note: Metabolites were putatively annotated based on exact mass matching (mass error < 5 ppm) and MS/MS spectral matching with public databases. Values are semi-quantitative estimates calculated by peak area normalization.
